# Cardiovascular Complications of Seasonal Influenza in the Pre- and Post-COVID-19 Era: Epidemiology, Mechanisms, and Clinical Implications

**DOI:** 10.3390/medsci14010057

**Published:** 2026-01-23

**Authors:** Chikodili Nora Nebuwa, Chukwudi Kingsley Orjichukwu, Rita Ogochukwu Orjichukwu, Peter Kanayochukwu Akpunonu, Paul Chikwado Ugwu, Somtochukwu Godfrey Nnabuife

**Affiliations:** 1 Nuvance Health, Vassar Brothers Medical Center, 45 Reade Place, Poughkeepsie, NY 12601, USA; 2 Well Health Manitoba Clinic, 790 Sherbrook Street, Winnipeg, MB R3A 1M4, Canada; 3 Medway Maritime Hospital, Gillingham ME7 5NY, UK; 4 North London NHS Foundation Trust, IT Department, 3rd Floor, West Wing, St Pancras Hospital, 4 St Pancras Way, London NW1 0PE, UK; 5 King George Hospital, Barley Lane, Ilford, Essex IG3 8YB, UK; 6 University of Wolverhampton, Wulfruna Street, Wolverhampton, West Midlands WV1 1LY, UK

**Keywords:** COVID-19, influenza, public health interventions, cardiovascular complications, myocardial infarction

## Abstract

Influenza has long been a well-documented contributor to cardiovascular morbidity and mortality, particularly among high-risk groups. COVID-19 has notably altered the seasonality and natural history of pandemic influenza, with broad implications for related cardiac complications. This review examines the interaction between influenza and cardiovascular illness, especially myocardial infarction, congestive heart failure, stroke, and other acute cardiac events. We review the impact of the COVID-19 pandemic on influenza transmission dynamics, public health policy, and the evolving burden of cardiovascular complications. New evidence indicates that both diseases exacerbate endothelial dysfunction, systemic inflammation, and prothrombotic states, thereby increasing cardiovascular risk. A comparative analysis of pre- and post-COVID-19 influenza-related cardiac complications clarifies evolving trends and guides future preventive strategies. In light of the recent resurgence of influenza following the relaxation of COVID-19 mitigation measures, maximizing vaccine coverage and collaborating to manage viral infections in patients with cardiovascular disease are critical. This review focuses on key research needs to understand long-term cardiac consequences and the urgent requirement for targeted public health strategies to counter viral-mediated cardiovascular threats. In the post-COVID era, integrating influenza and COVID-19 vaccination strategies into cardiovascular risk management may represent a critical opportunity to reduce virus-triggered cardiovascular morbidity and mortality.

## 1. Introduction

Orthomyxoviruses cause influenza every year, resulting in millions of people worldwide developing the illness [1]. Patients with this condition typically present with fever, cough, and sore throat, among other symptoms. Influenza primarily affects the respiratory system but also causes widespread effects throughout the body, including cardiovascular complications. Research indicates that people with flu face a higher risk of heart attacks and death from cardiovascular diseases [2].

In addition to influenza, SARS-CoV-2 infection has emerged as a major viral trigger of cardiovascular injury, with both viruses sharing pathophysiological pathways that link acute respiratory infection to cardiovascular events [3]. Although influenza has historically been the most studied viral precipitant of myocardial infarction, stroke, and heart failure exacerbations, accumulating evidence shows that COVID-19 induces comparable cardiovascular alterations through systemic inflammation, endothelial dysfunction, and prothrombotic states. Both influenza viruses and SARS-CoV-2 activate innate immune responses characterized by cytokine release (including interleukin-6 and tumour necrosis factor-α), endothelial activation, platelet aggregation, and disruption of vascular homeostasis [4], thereby increasing the risk of ACSs, arrhythmias, and cerebrovascular events. While SARS-CoV-2 exhibits a greater propensity for direct endothelial infection and prolonged cardiovascular sequelae, influenza-associated cardiovascular risk is well known to peak in the acute phase of infection, particularly within the first week [5]. Recognizing these shared yet distinct mechanisms is essential for understanding how respiratory viral infections precipitate cardiovascular events across different epidemiological eras and for providing a rationale for integrated preventive strategies, including vaccination and early antiviral therapy, to mitigate virus-triggered cardiovascular morbidity and mortality.

The integration of both seasonal influenza and SARS-CoV-2 within a unified narrative framework is deliberate and conceptually grounded rather than coincidental. Although these viruses differ in virological characteristics, transmission dynamics, and clinical progression, they constitute the two most significant respiratory viral threats to cardiovascular health in the contemporary era [1]. Both infections induce acute cardiovascular events via analogous mechanisms, encompassing systemic inflammation, endothelial dysfunction, platelet activation, and prothrombotic states. Conversely, the COVID-19 pandemic has altered influenza transmission patterns, immunity development, healthcare delivery, and cardiovascular risk assessment. Therefore, analysing influenza-associated cardiovascular complications alone without considering the disruptive and residual cardiovascular impacts of SARS-CoV-2 [2] would yield an incomplete and historically fragmented understanding. An integrated framework facilitates direct comparison of shared and distinct pathophysiological mechanisms, elucidates how pandemic-era public health interventions have reshaped influenza-related cardiac risk, and informs comprehensive prevention strategies, particularly vaccination and early antiviral therapy, in the post-COVID era [3].

During pandemic periods, the United Kingdom experiences increased healthcare needs related to the respiratory system. In the 2023–2024 period, hospitals in England recorded 868,212 emergency admissions for respiratory diseases, reflecting a significant rise in cases [6]. Moreover, doctors’ practices report an increase in consultations for respiratory tract infections during the winter months.

Flu epidemics cause 10,500 to 25,000 deaths each year in England and Wales. Worldwide, the flu season causes 290,000 to 650,000 respiratory deaths per year. There have been several influenza pandemics throughout history, the most deadly being the Spanish flu in 1918–1919, when approximately 50 million people died worldwide [7]. In 2009, the H1N1 virus emerged and caused a large number of cases, but the death rate was lower than in previous pandemics. In England, there were roughly 540,000 symptomatic H1N1 cases, with a case fatality rate of about 26 deaths per 100,000 cases [8]. The rapid transmissibility of viral variants contributed to widespread infection, necessitating accelerated vaccination programs and enhanced public health preparedness.

The UK has experienced major flu outbreaks in recent years. Since 2022, at least 18,000 deaths in England have been associated with the flu, prompting health experts to recommend that all eligible individuals receive antiviral therapy, including the season’s free NHS vaccination [9]. NHS officials remain steadfast in their commitment to immunization and public health measures to mitigate and control the burden of influenza and other respiratory infections.

In the United States, an estimated 36,000 deaths occur annually from influenza infection [10], and the morbidity–mortality impact remains higher during particular epidemic seasons and in the initial several years of pandemic influenza virus circulation [11]. Preparation for the next influenza pandemic, including anxiety about the current zoonotic infections caused by highly pathogenic avian IAV of subtype H5N1 [12], has, in most respects, been informed by the worst flu virus epidemic in history, the notorious ‘Spanish’ influenza pandemic of 1918–1919 [13].

Epidemiological and clinical research have well documented the association of influenza with cardiovascular complications. Influenza-related inflammation and coagulation abnormalities may result in acute cardiac events, assuming predisposing heart conditions are present [4]. Given that influenza-related cardiovascular morbidity represents a significant burden on healthcare resources, increased awareness and research into these complications are clearly required. If adverse outcomes related to early diagnosis and intervention can be mitigated, then incorporating cardiovascular considerations into influenza management protocols may alleviate them [5].

The COVID-19 pandemic has significantly affected the epidemiology and clinical course of influenza, as well as the associated cardiac problems [14]. Viral transmission dynamics have altered the impact and prevalence of influenza, the effects of mask use and physical distancing on public health measures, and changes in healthcare resource allocation. In addition, SARS-CoV-2 and influenza co-infections increase risk, making clinical management of cardiac conditions challenging [6,15]. A comparative analysis of pre- and post-COVID-19 influenza seasons will be valuable for understanding changing trends in viral infections and their cardiovascular impact [16].

In the pre-COVID-19 era, seasonal influenza caused substantial cardiovascular morbidity and mortality. The history shows a higher incidence of acute myocardial infarction, heart failure, and arrhythmias during peak influenza seasons [6]. The pathogenesis underlying these associations involves direct viral invasion of myocardial tissue, amplified systemic inflammation, and enhanced platelet aggregation, leading to thrombotic complications [5,17]. Understanding trends and challenges in influenza-related cardiac complications before the COVID-19 pandemic provides a basis for evaluating the post-pandemic landscape and guiding future prevention strategies [15].

**Figure 1:**
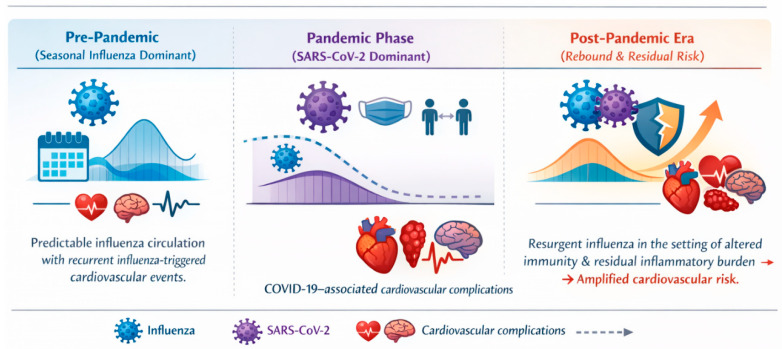
Conceptual framework illustrating the evolving relationship among seasonal influenza, COVID-19, and cardiovascular complications across the pre-pandemic, pandemic, and post-pandemic eras. In the pre-pandemic period, predictable seasonal influenza circulation is associated with recurrent influenza-triggered cardiovascular events. During the COVID-19 pandemic, although non-pharmaceutical interventions markedly suppressed influenza transmission, SARS-CoV-2 infection independently contributed to cardiovascular complications, including myocardial injury, myocarditis, thromboembolic events, arrhythmias, and stroke, as indicated by dedicated cardiovascular icon annotations. In the post-pandemic era, the resurgence of influenza amid altered population immunity and residual inflammatory burden is associated with renewed and potentially amplified cardiovascular risk.

This is an extensive review of cardiac complications of influenza, both before and after the COVID-19 era, highlighting significant changes in epidemiological trends [18], clinical presentation, and healthcare practice (see Figure 1). The review’s novelty lies in its comparative approach, examining pre-pandemic influenza trends alongside post-COVID alterations in viral spread, immune response, and cardiovascular presentation [16]. This research aims to inform our understanding of the link between respiratory illness and cardiovascular disease [19] and the potential implications for health policy and clinical practice by synthesizing readily accessible evidence and clinical experience.

To avoid redundancy and enhance clarity, this review adopts a thematic structure in which mechanistic pathways linking influenza to cardiovascular complications are discussed in a dedicated section and then referenced rather than restated in the epidemiological and clinical sections. Distinctions among the pre-COVID-19, pandemic, and post-COVID-19 eras are addressed in clearly demarcated subsections to facilitate continuity and chronological coherence.

The organization of this paper is as follows. Section 2 outlines the methods and literature search strategy. Section 3 examines the epidemiology and pathophysiological mechanisms linking seasonal influenza to cardiovascular complications, including myocardial infarction, stroke, heart failure, and cardiovascular mortality. Section 4 specifically addresses the impact of the COVID-19 pandemic on influenza epidemiology and cardiovascular disease, including changes in transmission patterns, long-term cardiac sequelae of SARS-CoV-2 infection, co-infection with influenza, and vaccination-related cardiovascular implications in the post-COVID era. Section 5 discusses the long-term cardiovascular consequences of influenza and COVID-19, including post-viral syndromes and comparative outcomes. Section 6 summarises key findings, clinical and public health implications, research gaps, and future directions.

## 2. Methods: Literature Search Strategy

This narrative review was conducted using a structured and comprehensive literature search to identify relevant studies examining the cardiovascular complications of seasonal influenza, with particular emphasis on comparisons between the pre-COVID-19 and post-COVID-19 eras. Electronic databases, including PubMed/MEDLINE, Scopus, Web of Science, and Google Scholar, were systematically searched for articles published between January 2000 and March 2025.

The search strategy combined Medical Subject Headings (MeSH) terms and free-text keywords, including but not limited to, the following: “influenza”, “seasonal influenza”, “cardiovascular complications”, “myocardial infarction”, “stroke”, “heart failure”, “myocarditis”, “COVID-19”, “SARS-CoV-2”, “co-infection”, and “vaccination”. Boolean operators (AND/OR) were applied to refine search results.

Eligible studies included observational studies, randomized controlled trials, meta-analyses, systematic reviews, surveillance reports, and extensive population-based cohort studies that investigated epidemiology, mechanisms, clinical outcomes, or preventive strategies related to influenza-associated cardiovascular disease (CVD). Articles focusing exclusively on non-cardiovascular outcomes, animal-only studies without clinical correlation, conference abstracts without full text, and non-English publications were excluded.

Additional relevant articles were identified through manual screening of reference lists from key publications and recent reviews. The selected literature was synthesised qualitatively to provide an integrated overview of epidemiological trends, pathophysiological mechanisms, clinical management, and preventive strategies related to influenza- and COVID-19-associated cardiovascular complications.

**Figure 2:**
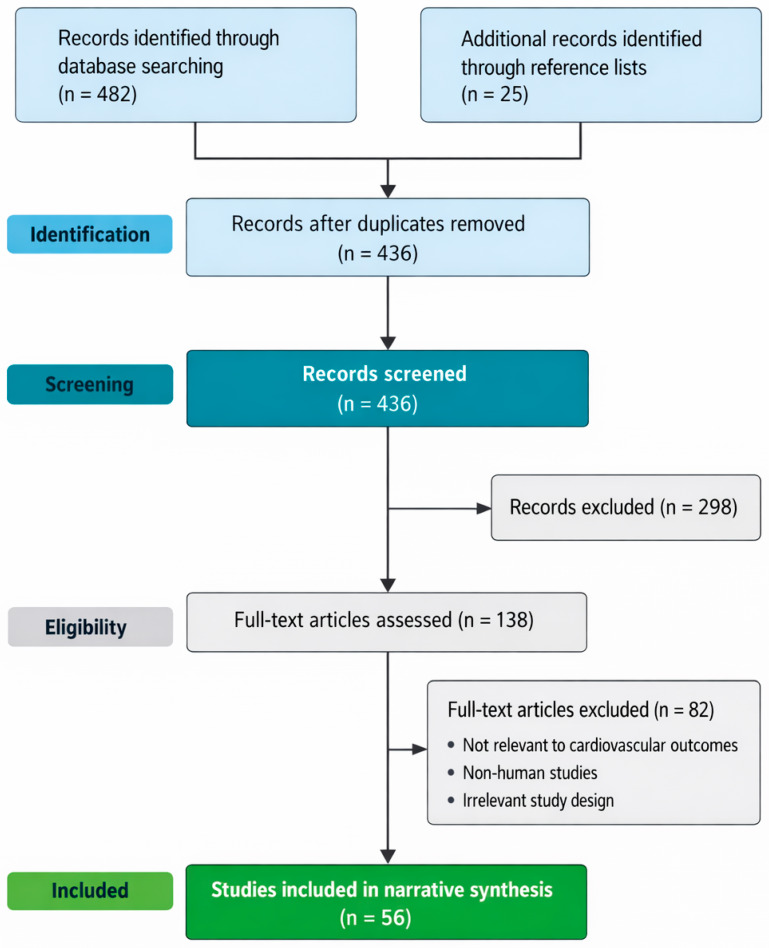
PRISMA-style flow diagram illustrating the identification, screening, eligibility assessment, and inclusion of studies for this narrative review on influenza- and COVID-19-associated cardiovascular complications.

The literature selection process followed a transparent, structured approach adapted from the PRISMA (Preferred Reporting Items for Systematic Reviews and Meta-Analyses) framework, tailored for a narrative review design. Database searches initially identified records across PubMed/MEDLINE, Scopus, Web of Science, and Google Scholar. After removal of duplicate records, titles and abstracts were screened for relevance to influenza- and COVID-19-associated cardiovascular outcomes. Full-text articles were subsequently assessed for eligibility based on predefined inclusion and exclusion criteria, including relevance to cardiovascular complications, human clinical data, and applicability to pre- and post-COVID-19 contexts. Additional studies were identified through manual screening of the reference lists of key publications. A PRISMA-style flow diagram summarises the identification, screening, eligibility, and inclusion stages of the literature selection process (Figure 2). This diagram is intended to enhance transparency in study selection and to acknowledge the narrative synthesis approach employed in this review.

The majority of evidence linking influenza and SARS-CoV-2 infections to cardiovascular outcomes derives from observational, epidemiological, and self-controlled case series studies. While these designs provide strong temporal associations and biologically plausible links, they do not establish definitive causality. Accordingly, associations described in this review should be interpreted as indicative of increased risk rather than proof of direct causation. Mechanistic pathways discussed are supported by experimental, translational, and clinical observations but remain inferential in humans.

To support temporal interpretability, the included studies were evaluated based on their primary data collection period. Evidence generated before 2020 was classified as pre-COVID-19, while studies incorporating data from 2020 onward were considered post-COVID-19. Conclusions in this review are accordingly stratified by era, with established pre-pandemic associations presented separately from post-pandemic observational trends influenced by COVID-19-related public health measures and healthcare system changes.

Mechanistic insights discussed in this review are derived from an integrated synthesis of experimental models, translational studies, and human clinical evidence. While experimental and in vitro studies provide biological plausibility regarding inflammatory, endothelial, and thrombotic pathways, extrapolation to human cardiovascular outcomes is primarily supported by convergent findings from epidemiological analyses, self-controlled case series, biomarker studies, and large population-based cohorts. Accordingly, mechanistic pathways are presented as biologically plausible contributors rather than definitive causal explanations and are interpreted within the context of human clinical and observational data. This triangulation approach reflects current best practice in cardiovascular translational research, particularly for infection-triggered acute cardiovascular events, where randomized mechanistic trials in humans are not feasible.

## 3. Epidemiology, Mechanisms, and Cardiovascular Impact of Influenza

### 3.1. Epidemiology of Influenza and Cardiovascular Risk

Nearly 25% of deaths in the US and 30% of deaths worldwide are attributable to CVD [6]. Apart from the conventional risk factors (such as smoking, high blood pressure, diabetes, obesity, sedentary lifestyles, and dyslipidemia), influenza illness has been increasingly recognised as an essential trigger and risk factor for cardiovascular morbidity and mortality [7]. Influenza virus RNA has been detected in human atherosclerotic plaques, and infectious agents have been implicated in the development of atherosclerosis [8]. Epidemiologic and experimental data reveal that influenza infections result in direct cardiac changes (from myopericarditis [9] to acute myocardial infarction [10]), systemic responses, and population-level increases in cardiovascular hospitalizations and deaths. Influenza epidemics are consistently associated with increases in major cardiovascular events, including acute myocardial infarction, stroke, heart failure exacerbation, and cardiovascular mortality (Table 1).

Influenza epidemics occur each winter in temperate countries and coincide with increases in CVD-related deaths. These predictable time features can guide public health response and mitigation strategies. Weeks in advance, the timing of the seasonal influenza peak can be predicted for municipalities. Since inflammatory cytokines and prothrombotic changes are associated with seasonal influenza, which is related to cardiovascular disease mortality [23], accurate and reliable influenza forecasts could help predict the occurrence and magnitude of winter spikes in cardiovascular mortality.

Table 1 summarises the major cardiovascular complications associated with influenza infection, highlighting the strength of the epidemiological evidence, the magnitude of risk, and the underlying biological mechanisms. Acute myocardial infarction and ischaemic stroke demonstrate the strongest temporal associations, particularly within the first week following infection. Notably, the table also illustrates the protective effect of influenza vaccination against major adverse cardiovascular events.

Beyond the magnitude of risk, the timing of cardiovascular events following influenza infection is critical for clinical decision-making.

Assessment of mechanistic evidence in humans relies on integrated clinical and biomarker-based proxies rather than direct plaque-level assays [24]. In human studies, plaque destabilisation and thrombosis following influenza infection are inferred through convergent evidence, including acute elevations in inflammatory biomarkers (e.g., C-reactive protein, interleukin-6), endothelial activation markers (e.g., von Willebrand factor), platelet activation indices, and coagulation parameters such as fibrinogen and D-dimer. These biomarkers are temporally associated with laboratory-confirmed influenza and coincide with sharply increased risks of acute myocardial infarction and ischaemic stroke in self-controlled case series and population-based cohorts [11]. Advanced vascular imaging studies and post-mortem analyses further support infection-associated plaque vulnerability, although direct intraplaque mechanistic assays in living humans are not ethically or clinically feasible during acute infection. Accordingly, mechanistic pathways linking influenza to plaque destabilisation and thrombosis in humans should be interpreted as biologically plausible, indirectly supported, and clinically validated through consistent temporal and outcome-based associations rather than definitive causal proof [25].

Interpretation of Heterogeneity Across Study Designs.

Reported associations between influenza infection and cardiovascular outcomes vary substantially across studies [7,15], in part due to differences in epidemiological design, exposure definition, confounding control, and outcome ascertainment. Self-controlled case series (SCCS) studies consistently demonstrate the strongest short-term associations, often reporting a five- to six-fold increase in acute myocardial infarction risk within the first week of laboratory-confirmed influenza, mainly because this design inherently controls for fixed individual-level confounders and precisely defines risk windows [26].

In contrast, traditional cohort and case–control studies tend to report smaller effect sizes, reflecting residual confounding from comorbidities, health-seeking behaviour, vaccination status, and socioeconomic factors that are incompletely captured in administrative datasets. Ecological and time-series analyses are valuable for demonstrating population-level temporal correlations between influenza activity and cardiovascular mortality. Still, they are particularly vulnerable to exposure misclassification and environmental fallacy, which may attenuate or inflate observed associations [23].

Importantly, variation in cardiovascular outcomes also reflects differences in endpoint definitions (e.g., incident versus recurrent myocardial infarction, hospitalisation versus mortality), diagnostic confirmation of influenza (laboratory-confirmed versus clinically diagnosed), and length of follow-up. Collectively, these methodological differences explain much of the heterogeneity in reported risk estimates and underscore the importance of interpreting influenza-associated cardiovascular risk within the context of study design rather than as a single uniform effect size [25,27].

#### 3.1.1. Influenza and Myocardial Infarction (MI)

**Figure 3:**
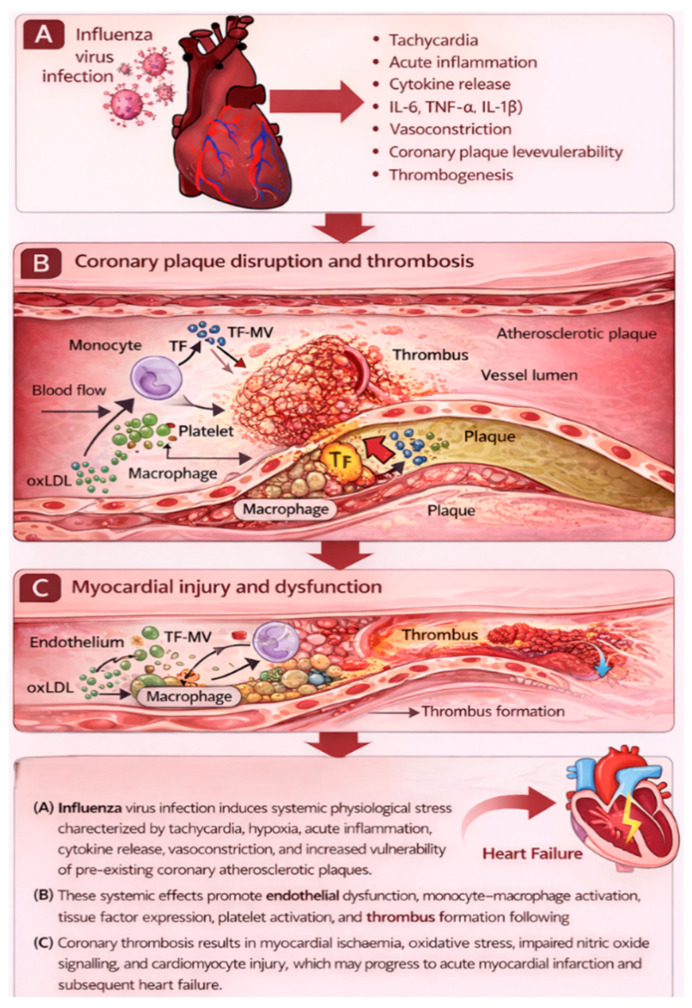
Pathophysiological cascade linking influenza infection to acute myocardial infarction.

AMI and other ischemic vascular events may be caused by influenza infection, a theory gaining attention [28]. This causal link has significant implications for both primary (influenza vaccination) and secondary (antiviral therapy or antithrombotic prophylaxis) preventive measures. Because conventional epidemiologic designs, such as cohort, case–control, and ecological studies, have inherent limitations, proving this link convincingly is difficult. An additional design, the self-controlled case series (SCCS), offers significant advantages over conventional designs because it removes fixed confounder bias by making each participant their own control, and it is especially well-suited to explore associations where the exposure (influenza infection) and outcome (AMI) are relatively uncommon [17]. This technique was recently used for the first time to investigate the association between influenza illness and AMI in Ontario, Canada. Case series were created by integrating multiple health registers. Warren-Gash et al. reported that laboratory-confirmed influenza infection increased the risk of acute myocardial infarction by approximately six-fold within the first week after infection [29]. This suggests that influenza virus infection-specific effects may contribute to an increased short-term risk of AMI, with significant implications for influenza prevention and therapy in cardiovascular risk management [30]. However, the results must be verified before guidelines and regulations may be altered. The biological mechanisms linking acute influenza infection to myocardial infarction involve a cascade of inflammatory, endothelial, and thrombotic processes, as illustrated in Figure 3. Specifically, the terminal stage of this cascade involves plaque rupture with superimposed thrombus formation, leading to acute coronary occlusion and clinical myocardial infarction.

Influenza infection triggers systemic inflammation characterised by cytokine release, endothelial dysfunction, and platelet activation. These processes destabilise atherosclerotic plaques, promote thrombosis, and increase myocardial oxygen demand, culminating in acute coronary occlusion and myocardial infarction.

#### 3.1.2. Influenza and Stroke Incidence

Stroke is a key contributor to morbidity and mortality, with health service costs, excluding social or economic costs, estimated at £2.8 billion annually in the UK [29]. Hypertension and family history of stroke, both well-established risk factors, are present in only 50–60% of patients with ischaemic stroke, suggesting that additional acute triggers such as systemic infection and inflammation may contribute to stroke onset [29,31].

A systematic review of the potential triggers of ischaemic stroke concludes that infection, including respiratory disease, constitutes a potential trigger [32]. There was a significant association between ischaemic stroke and illness within the previous week (OR = 2.91; 95% CI, 1.41 to 6.00) or month (OR = 2.41; 95% CI, 1.78 to 3.27) [32]. Influenza has been particularly implicated, with a documented tripling of the influenza rate corresponding to about a 6% change in stroke occurrence rate [33].

Antibiotics are ineffective in stroke; rather, the focus should be on the effectiveness of antiviral drugs for the strain of influenza virus one has. The vaccine’s contribution to reducing the chances of strokes has been acknowledged in some studies. However, the possibility of bias exists, which could distort the results due to confusion caused by factors that were not measured. This distortion stems in part from the belief that healthy people are more willing to get the vaccine. In contrast, riskier groups are less likely to act, making it difficult for them to get vaccinated.

The influenza vaccination has been associated with a lower risk of stroke in several observational studies, although residual confounding cannot be excluded. Influenza vaccination has been shown to correlate with a lower risk of stroke in various studies, as have other vaccines, with and without pneumococcal [34]. However, some studies [35,36,37,38] have shown no correlation, highlighting ongoing uncertainty regarding the magnitude and consistency of stroke-specific protection. Taken together, the apparently conflicting findings regarding influenza vaccination and stroke risk likely reflect significant methodological and population-level differences rather than actual biological inconsistency. Observational studies reporting protective associations are susceptible to healthy-user bias, differential healthcare access, and residual confounding, particularly as individuals who receive vaccination are often more health-conscious and better managed for vascular risk factors [39]. Conversely, studies demonstrating null associations frequently involve shorter follow-up periods, heterogeneous stroke phenotypes, or insufficient statistical power to detect modest risk reductions. Importantly, the absence of a consistent stroke-specific signal does not negate the broader cardiovascular benefits of influenza vaccination, which are robustly supported for myocardial infarction, heart failure exacerbations, and cardiovascular mortality. From a clinical and public health perspective, the totality of evidence supports influenza vaccination as a low-risk intervention with probable cerebrovascular benefit in high-risk populations, even if stroke-specific risk reduction cannot be quantified with certainty across all study designs [40].

Stroke is a multifactorial and clinically heterogeneous condition; therefore, attributing causality to a single trigger requires caution. Residual confounding, selection bias, and unmeasured behavioural or clinical factors may influence observational associations between influenza, vaccination status, and stroke risk.

**Figure 4:**
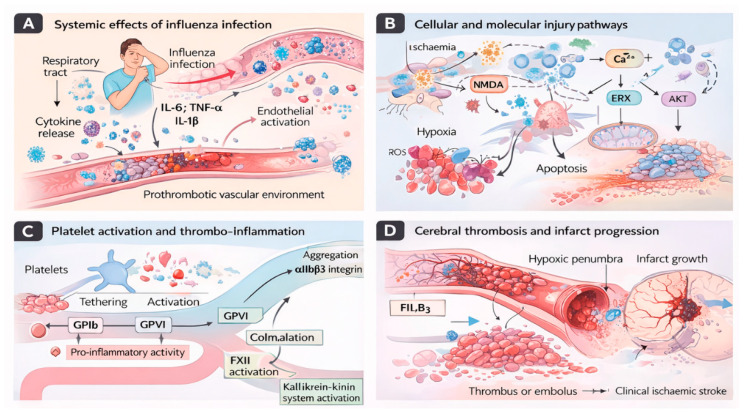
Mechanisms linking influenza infection to ischaemic stroke. (**A**) Influenza infection initiates a systemic inflammatory response characterised by cytokine release (IL-6, TNF-α, IL-1β), endothelial activation, and circulating immune cell recruitment, creating a prothrombotic vascular environment. (**B**) At the cellular level, hypoxia, oxidative stress, calcium dysregulation, and excitotoxic signalling activate molecular injury pathways involving NMDA receptor stimulation, ER stress, AKT signalling disruption, mitochondrial dysfunction, and apoptosis. (**C**) Concurrently, systemic inflammation promotes platelet tethering and activation via glycoprotein receptors (GPIb and GPVI), leading to platelet aggregation through αIIbβ3 integrin signalling and amplification of thrombo-inflammatory cascades through coagulation and kallikrein–kinin system activation. (**D**) These converging mechanisms facilitate cerebral thrombus or embolus formation, impair microvascular perfusion, and drive infarct expansion within the hypoxic penumbra, ultimately contributing to clinical ischaemic stroke.

Respiratory infections such as influenza may act as acute triggers for ischaemic stroke through inflammatory and prothrombotic mechanisms, as summarised in Figure 4.

#### 3.1.3. Cardiovascular Mortality and Influenza

Contagious influenza is a viral infection that significantly impacts public health, especially among patients with existing CVD [2]. Epidemiological studies consistently show an increase in cardiovascular deaths during periods of influenza [41], underscoring the link between viral infections and acute cardiovascular events. The primary mechanism is infection-induced inflammation associated with the flu, which exacerbates systemic CVD and leads to adverse outcomes such as heart attacks [41], worsening of congestive heart failure, and strokes. Every year, high-risk populations, such as those with CVD, are advised to get vaccinated against influenza due to its established preventive efficacy [42]. However, the specific CVD outcomes affected by seasonal influenza vaccination remain uncertain. In particular, those with pre-existing CVD often struggle to receive vaccinations [1]. Estimates of influenza VCR in patients with heart failure provide a stark example, with Asia reporting around 0% coverage and Europe around 80% [43]. Only 2% of the general population in China is estimated to receive the influenza vaccine, with even lower numbers among high-risk groups [44]. Among seniors aged 65 years and older who are eligible for free vaccination, VCR is approximately 20% [45].

Multiple studies focusing on specific populations have shown a close link between influenza outbreaks and increases in CVD mortality. For example, during the 1918 influenza pandemic, there was an apparent rise in deaths attributed to heart disease. Similar increases were observed during subsequent seasonal flu epidemics. These trends have been confirmed more recently. Ref. [46] found a higher incidence of cardiovascular mortality associated with flu activity, particularly in elderly patients or those with pre-existing coronary artery disease. In another well-known study, the authors found that the rate of heart attacks rose sixfold during the first week after confirmed influenza infection, further establishing the relationship between influenza and acute heart problems. The cardiovascular mortality following influenza infection is multifactorial, reflecting the combined effects of systemic inflammation, endothelial dysfunction, prothrombotic states, and increased myocardial oxygen demand.

Influenza infection triggers a robust inflammatory response, leading to the release of interleukin-6 (IL-6) and tumor necrosis factor-α (TNF-α), which contribute to endothelial dysfunction, plaque destabilization, and a hypercoagulable state that predisposes to acute coronary syndromes and cardiovascular mortality [37,45,47]. In addition, fever-induced tachycardia and increased myocardial oxygen demand further exacerbate myocardial ischemia in patients with limited coronary reserve [45,48]. Furthermore, several studies have shown that viral infections can induce arrhythmias and worsen the heart’s ability to pump blood in already susceptible patients.

Influenza infection in patients with known cardiovascular comorbidity is associated with increased mortality, and therefore, measures such as annual influenza vaccination are necessary to mitigate this risk [27]. A meta-analysis of clinical trials found that yearly influenza vaccination reduces major adverse cardiovascular events by 34%, with a greater reduction among patients with recent ACS [49]. However, despite this evidence, a substantial number of patients with cardiovascular disorders do not receive vaccination, necessitating stronger public health programs to emphasize the importance of immunization.

Influenza is a major contributor to mortality from CVDs, especially among people who already have heart disease. The inflammatory and thrombotic effects of influenza infection worsen cardiovascular illness, increasing the risk of myocardial infarction [20], heart failure, and stroke. Given these dangers, the influenza vaccine needs to be prioritized as a critical preventive intervention for reducing cardiovascular adverse effects and mortality during pandemic influenza [15]. Additional research is required to examine other therapeutic interventions for reducing the cardiovascular consequences of influenza illness.

**Figure 5:**
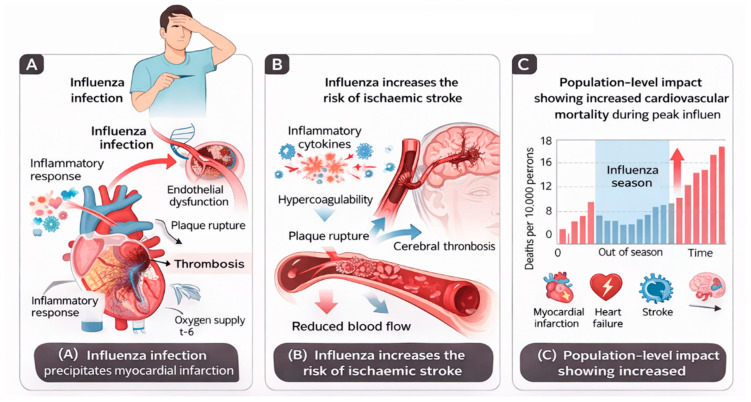
Cardiovascular complications of influenza infection. (**A**) Acute influenza infection precipitates myocardial infarction through systemic inflammation, endothelial dysfunction, plaque destabilisation, and thrombosis. (**B**) Influenza infection increases the risk of ischaemic stroke via inflammatory cytokine release, endothelial injury, and hypercoagulable states, leading to cerebral thrombosis. (**C**) Population-level impact of influenza infection showing increased cardiovascular mortality during peak influenza activity, driven by excess myocardial infarction, heart failure exacerbations, and stroke. Proposed mechanisms are derived from observational human studies and experimental data and should not be interpreted as definitive causal pathways.

At the population level, these acute cardiovascular events contribute to excess cardiovascular mortality during peak influenza activity, as shown in Figure 5.

### 3.2. Preventive Measures for Influenza and SARS-CoV-2

The most effective and economical way to prevent influenza is the seasonal flu vaccine. Although vaccine efficacy may vary by population, recent research indicates that the influenza vaccine reduces the risk of flu by 40–60% during peak seasons [50]. During the 2017–2018 season, pre-season vaccination helped reduce an estimated 3.2 million flu-related medical visits, 91,000 flu-related hospitalizations, and 5700 flu-related fatalities [50]. Nearly all currently available influenza vaccines are safe and well tolerated, with rare and often modest adverse effects [51].

The influenza vaccine has been linked to a reduction in major adverse cardiovascular events, in addition to preventing the virus [49]. A meta-analysis by Udell et al. [49] found that the influenza vaccine was associated with a lower risk of composite cardiovascular events (2.9% vs. 4.7%; RR, 0.64 [95% CI, 0.48–0.86], *p* = 0.003). Given the link between the influenza virus and an elevated risk of MI, the potential preventive effect of the influenza vaccine in reducing adverse cardiovascular events is critical in clinical practice.

Since late 2020, multiple COVID-19 vaccines have completed large-scale Phase III clinical trials and have been authorized for widespread global use. Extensive post-marketing surveillance and real-world evidence now confirm that COVID-19 vaccines are safe and effective and are associated with a substantial reduction in severe disease, hospitalization, mortality, and virus-related cardiovascular complications. Initial apprehensions during the early stages of vaccine development, including immune enhancement and harmful inflammatory responses, were largely theoretical and based on preclinical or preliminary coronavirus vaccine platforms. These concerns have not been substantiated in large human populations following mass vaccination campaigns. On the contrary, COVID-19 vaccination has been shown to significantly reduce systemic inflammation, endothelial injury, and thromboembolic risk associated with SARS-CoV-2 infection, particularly in individuals with pre-existing cardiovascular disease. Consequently, COVID-19 vaccination now represents a cornerstone of cardiovascular risk mitigation in the post-pandemic era, alongside established influenza vaccination strategies [15,52].

References to early vaccine development challenges in this review reflect historical contexts and do not contradict the established safety and clinical effectiveness of currently authorised COVID-19 vaccines.

Given that influenza vaccine clinical trials began in the mid-1930s and that the first bivalent vaccine demonstrating adequate protection against flu outbreaks became available in December 1942 [53], the development of an effective COVID-19 vaccine may take years. Research into Middle East respiratory disease (MERS) and SARS may help accelerate the development of a possible vaccine. The best defences against SARS-CoV-2 transmission until a vaccine is developed are facemask use, hand hygiene, and physical distancing. Wearing face masks has been shown to effectively reduce the number of virus particles in respiratory droplets. According to [54]’s study, if up to half the population wears face masks regularly, the R value can fall below 1.0, eliminating the risk of another wave [55]. These precautions also significantly reduce the risk of influenza transmission. By implementing facemask use at the population level, the risk of an influenza pandemic can be reduced by lowering the influenza infection attack rate (Rint) below 1.0 [55]. Adequate adherence to these measures is therefore critical to delaying or limiting the risk of a dual pandemic.

While this is purely hypothetical, if the COVID-19 vaccine is as effective as the influenza vaccine, it may also reduce cardiovascular risk by suppressing acute inflammatory and procoagulant stimuli. This process is thought to prevent changes in endothelial function and the weakening of vulnerable atherosclerotic plaques, which may lead to coronary artery occlusion [56]. Individuals with underlying CVD who are vulnerable to recurrent cardiovascular events as a result of COVID-19 infection may benefit from this as a potential secondary preventive measure.

### 3.3. Mechanisms of Cardiac Involvement in Influenza

**Figure 6:**
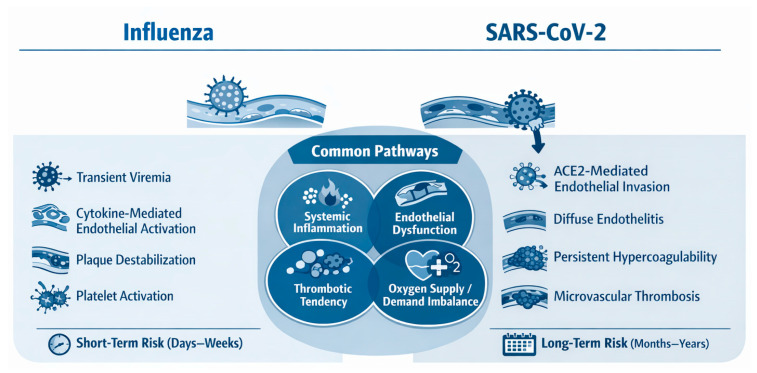
Shared and distinct mechanisms of cardiovascular injury induced by influenza virus and SARS-CoV-2.

CVD may be directly and indirectly affected by the flu virus. Some of these effects are due to the virus’s ability to attach to myocardial cells (heart muscle) and damage them through the immune system’s response to the virus, as well as through its effects on blood vessels [57]. These mechanisms increase the likelihood of acute cardiovascular events in individuals with preexisting heart disease; thus, identifying these pathways is critical for early diagnosis, evaluating cardiovascular risk, and providing appropriate therapies if needed. Although influenza and SARS-CoV-2 share inflammatory and thrombotic pathways, significant differences exist in the magnitude, duration, and vascular targets of cardiovascular injury, as illustrated in Figure 6.

Both the influenza virus and SARS-CoV-2 trigger systemic inflammation, endothelial dysfunction, and pro-thrombotic states that predispose to acute cardiovascular events. Influenza is typically associated with transient immune activation and short-term cardiovascular risk, whereas SARS-CoV-2 often causes direct endothelial injury, sustained hypercoagulability, and prolonged cardiovascular sequelae that extend beyond the acute infection period.

To better contextualise the cardiovascular effects of influenza in the post-pandemic era, it is helpful to compare its pathogenic mechanisms with those of SARS-CoV-2. Both viruses share overlapping inflammatory and thrombotic pathways, while also exhibiting distinct patterns of myocardial and endothelial involvement. Table 2 provides a comparative overview of these mechanisms and their cardiovascular consequences.

#### 3.3.1. Relative Contribution of Direct Myocardial Injury and Systemic Inflammation

Influenza-related cardiac injury arises through both direct myocardial involvement and indirect systemic mechanisms; however, available clinical and epidemiological evidence suggests that systemic inflammation represents the predominant pathway at the population level, whereas direct myocardial invasion is comparatively rare [25]. Histopathologically confirmed influenza myocarditis has been documented, but it occurs infrequently and primarily in severe or fatal cases. In contrast, the temporal clustering of myocardial infarction, stroke, and heart failure exacerbations within days of influenza infection strongly supports an indirect mechanism mediated by systemic inflammation, endothelial dysfunction, plaque destabilisation, and hypercoagulability. Cytokine-driven inflammatory responses, particularly involving interleukin-6 and tumour necrosis factor-α, amplify thrombotic risk and myocardial oxygen demand, thereby precipitating acute cardiovascular events in individuals with underlying atherosclerotic disease [35]. Importantly, current evidence does not permit precise quantitative attribution of risk between these pathways, as most data derive from observational studies rather than mechanistic human trials [25]. Consequently, influenza-related cardiac injury should be conceptualised as a spectrum on which systemic inflammatory mechanisms predominate, with direct myocardial involvement representing a less common but clinically significant manifestation.

#### 3.3.2. Viral Myocarditis and Endothelial Dysfunction

Influenza viruses can directly invade the heart muscle (myocardium), leading to myocarditis, or inflammation of the myocardium. However, these instances are not common in clinical practice. When the influenza virus enters cardiomyocytes (heart muscle cells) and endothelial cells (cells that line the interior of blood vessels), it causes inflammation, apoptosis, and increased water accumulation (oedema) [59]. This can manifest as symptoms of chest pain, arrhythmias, acute heart failure, or, in severe cases, cardiogenic shock. Histopathological findings indicate inflammatory infiltration and the presence of dead heart muscle (necrosis) in patients who have suffered from the consequences of influenza infection, which suggests that one mechanism of heart damage by influenza is through the direct killing effect on the heart muscle.

Endothelial dysfunction is the main pathway by which influenza infection causes cardiovascular complications. Endothelial cells are activated during influenza infection, leading to loss of normal vascular function, decreased nitric oxide availability, and vasoconstriction (narrowing) of the blood vessels. Dysfunctional endothelial cells develop an increased pro-adhesive and pro-thrombotic phenotype (attracting more platelets and white blood cells to aggregate on their surface). For patients with pre-existing atherosclerosis (hardening of the arteries), these changes will also increase the risk of plaque rupture, which can lead to ACS, and the risk of tissue loss due to reduced blood flow (hypoxia) in the heart [28].

Endothelial injury will also result in microvascular dysfunction. Microvascular dysfunction leads to a decreased myocardial perfusion reserve. It worsens myocardial ischaemia (insufficient blood supply) during periods of increased metabolic need, which is particularly relevant to older adults and patients with coronary artery disease. Even relatively minor endothelial perturbations may lead to clinically significant myocardial events in these patient groups [30].

#### 3.3.3. Role of Systemic Inflammation and Cytokine Storm

The most prominent feature of a severe case of influenza is a substantial systemic inflammatory response to infection. After the body’s innate immune response recognizes the virus, it triggers the release of several pro-inflammatory cytokines, including IL-6, TNF-α, and IL-1β. Severe cases are associated with a “massive inflammatory response,” more commonly referred to as a “cytokine storm,” characterized by elevated cytokine levels, widespread immune activation, and the development of multi-organ injury [60].

The role of inflammation in causing myocardial injury associated with influenza is well established. Increases in cytokine levels create unstable, thrombogenic plaques, activate the coagulation cascade, and induce a hypercoagulable state. Cytokines also depress myocardial contractility by impairing calcium handling, resulting in transient and/or sustained systolic dysfunction [47].

Inflammation-associated increases in heart rate and fever, along with increased metabolic (oxygen) demand on the myocardium, exacerbate the mismatch between oxygen supply and demand in patients with limited coronary reserves. This imbalance increases the risk of type 2 myocardial infarction and arrhythmias. Taken together, these inflammatory effects on the heart explain the clustering of acute myocardial infarction, stroke, and heart failure during and shortly after an influenza infection [61].

#### 3.3.4. Exacerbation of Pre-Existing Heart Disease

Patients with pre-existing CVD are often at high risk for suffering decompensation from an acute influenza infection. For anyone with heart failure, coronary artery disease, valvular heart disease, or arrhythmias, they have a high susceptibility to the haemo-dynamic and metabolic demands that an acute infection causes [62].

Acute influenza can cause fever, tachycardia, hypoxia, and changes in blood volume. These factors increase the heart’s workload, triggering symptoms of decompensated heart failure. Patients with low ejection fraction may develop inflammation-mediated myocardial contractile dysfunction and increased fluid retention during acute influenza infection, thereby increasing the risk of pulmonary congestion and hospitalization. Acute influenza also stimulates increased sympathetic nervous system activation, which in certain vulnerable patients can cause atrial fibrillation, ventricular arrhythmias, and sudden cardiac death [63].

Patients with coronary artery disease are at increased risk of acute plaque rupture and coronary thrombosis due to elevated systemic inflammation and pro-coagulative states induced by acute influenza infection. All three factors, increased myocardial oxygen consumption, endothelial dysfunction, and activated platelets, create an environment with an increased risk of adverse cardiovascular events, thus necessitating preventive measures in high-risk populations [63].

### 3.4. Management of Cardiac Complications During Influenza

Managing cardiac complications associated with influenza requires a holistic approach that addresses the viral aspect of the disease and its relationship to the heart. Early identification of the virus through accurate diagnostic testing, prompt initiation of effective antivirals, and improved cardiac management will be crucial to reducing overall morbidity and mortality, especially among persons at increased risk of developing cardiovascular problems related to influenza infection.

#### 3.4.1. Treatment Protocols for Influenza-Related Cardiac Events

Patients diagnosed with or suspected of having influenza who also exhibit cardiac symptoms should be evaluated and receive early cardiovascular specialist intervention. This evaluation may include some or all of the following tests: (1) ECG; (2) cardiac markers; (3) cardiac imaging, if clinically appropriate. A comprehensive assessment to differentiate among the following three major categories of disease is critical because treatment strategies for each category vary widely: viral myocarditis, ACS, and heart failure exacerbation [64].

At a minimum, all patients in one of the three categories above (viral myocarditis, ACS, and heart failure exacerbation) should be treated according to accepted medical practice guidelines, which include (1) use of antiplatelet agents (e.g., aspirin, clopidogrel, ticagrelor) and reperfusion therapy; (2) use of diuretics and neurohormonal antagonists for acute heart failure; and (3) provision of adequate rate or rhythm control, as needed for arrhythmias. Finally, for patients with severe anaphylaxis, including potential myopathy or cardiogenic shock, hemodynamic monitoring is strongly recommended [65].

Patients admitted to hospitals with influenza-related cardiovascular complications may benefit from a multidisciplinary team of specialists in cardiology, infectious disease, critical care, and related fields. Early intervention and assessment for ongoing deterioration in cardiac status, including admission to an intensive care unit (ICU) when clinically necessary, improve overall outcomes for this high-risk population.

#### 3.4.2. Antiviral and Anti-Inflammatory Approaches

Antiviral therapy is the primary treatment for influenza and will indirectly reduce the risk of cardiovascular issues by inhibiting viral replication and limiting systemic inflammation. The most effective neuraminidase inhibitors, including oseltamivir, should be started within 48 h of symptom onset; however, for those with severe influenza or hospitalized patients, there will still be some benefit after this initial 48 h period.

By reducing the viral load in the body, antiviral therapy lessens the intensity and duration of the inflammatory response, thereby lowering the risk of endothelial dysfunction, plaque destabilization, and myocardial damage. In individuals at high risk, observational studies indicate that early initiation of antiviral treatment is associated with reduced rates of cardiovascular events and increased survival [66].

Research into other anti-inflammatory treatments continues. The routine use of systemic corticosteroids in uncomplicated influenza patients is not recommended due to their potential adverse effects. Still, for selected patients with severe myocarditis or cytokine-mediated cardiac dysfunction, specific immunomodulatory therapies may be beneficial. Further studies are required to optimise the combination of controlling the virus and modulating the immune system to reduce the risk of cardiovascular complications from influenza [67].

Although this subsection focuses primarily on managing influenza-related cardiovascular complications, its clinical principles are particularly relevant in the post-COVID-19 era. SARS-CoV-2 infection has increased cardiovascular susceptibility through chronic endothelial dysfunction, systemic inflammation, and pro-thrombotic conditions. Consequently, patients presenting with influenza in the post-COVID period, especially those with prior SARS-CoV-2 infection or underlying cardiovascular disease, may be at increased risk of cardiac complications. Early recognition, prompt antiviral therapy, and guideline-directed cardiovascular management during influenza infection remain critical components of post-pandemic cardiovascular care.

## 4. Post-COVID Era: Impact of COVID-19 on Influenza and Cardiac Health

Having established the cardiovascular effects of influenza in the pre-pandemic era, the following section focuses explicitly on how COVID-19 altered influenza epidemiology and reshaped cardiovascular risk profiles associated with the virus in the post-pandemic period.

The global COVID-19 pandemic has had numerous indirect effects beyond the direct impact of SARS-CoV-2 infection [68]. The two primary effects are on influenza epidemiology and on cardiac disease burden. In this section, the effect of COVID-19 on influenza trends and cardiac complications in the post-pandemic era is presented for overview.

Public health measures instituted to contain COVID-19 in the early pandemic years (2020–2021) [69], including social distancing, mask mandates, and travel restrictions, were associated with a precipitous decline in influenza cases globally. Post-pandemic, influenza is returning with unpredictable seasonality and changed patterns of virus circulation. Increased susceptibility in subsequent seasons: Reduced exposure to circulating variants due to non-pharmaceutical intervention measures may have lowered population immunity to influenza viruses [70].

An international study using global influenza surveillance data found that influenza activity was historically low during the pandemic but rebounded with greater severity in some regions after COVID-19 restrictions were lifted [71]. These findings indicate that shifts in virus transmission dynamics and population immunity will necessitate future changes in influenza vaccination strategies.

COVID-19 has been associated with greater cardiovascular complications during acute infection and adverse health outcomes afterward. However, studies have reported increased incidence of pericarditis, thromboembolic, and myocarditis events following SARS-CoV-2 infection [36]. Even in patients with mild COVID-19, post-acute cardiovascular sequelae like arrhythmias, heart failure, and myocardial infarction have been demonstrated [37]. Recent studies show that patients after COVID-19 are at increased risk of developing CVDs for at least a year after infection [38]. This indicates that COVID-19 may have chronic consequences for heart health, and the long-term effects of the virus may require continuous monitoring and control of cardiovascular risks for recovered individuals.

**Figure 7:**
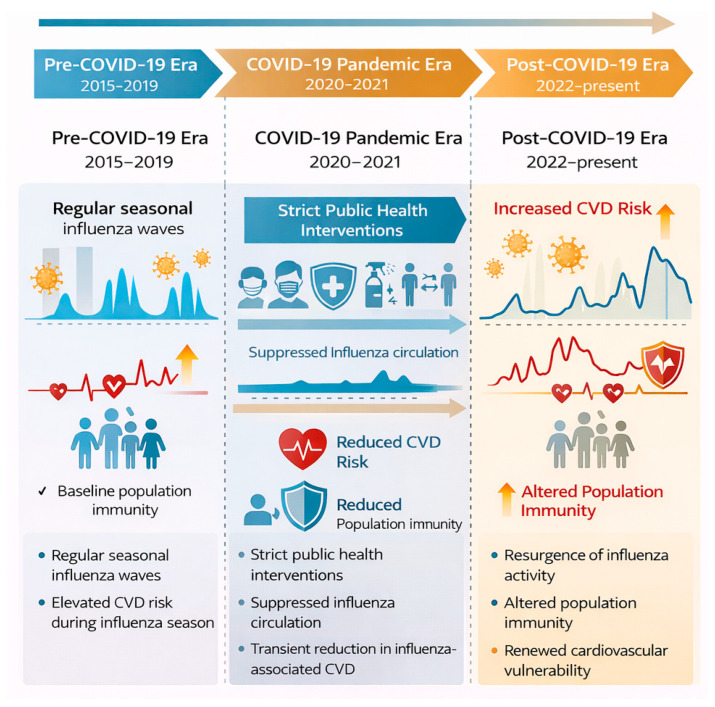
Evolution of influenza epidemiology and cardiovascular risk across the pre-COVID-19, pandemic, and post-COVID-19 eras. Public health interventions during the COVID-19 pandemic led to marked suppression of influenza circulation and a transient reduction in influenza-associated cardiovascular events. Post-pandemic resurgence of influenza, combined with altered population immunity, has been accompanied by renewed cardiovascular vulnerability.

Managing flu and cardiac health in the post-COVID era presents many challenges. The evolution of influenza circulation patterns also informs updated vaccination strategies. At the same time, the heightened cardiovascular risk following COVID-19 suggests that enhanced surveillance of this risk, as well as modified engagement with prevention programs, are required [72]. Long-term interactions among infectious diseases and risk-factor diseases associated with them are inevitable unless evidence-based policy interventions restrain these diseases [73]; thus, future research should explore and inform not only these fine-grained details but also the attitudes and behaviors surrounding such interventions. The COVID-19 pandemic has reshaped influenza circulation patterns and the associated cardiovascular burden across successive phases, as summarized in Figure 7.

### 4.1. Changes in Influenza Epidemiology Post-COVID

#### 4.1.1. Decline in Influenza Incidence During the Pandemic

During the COVID-19 pandemic, the implementation of aggressive public health policies led to an unexpected decline in the rate of influenza cases worldwide [41]. Data from the totality of global influenza surveillance systems indicated that in the 2020–2021 season, influenza activity was extremely low, with some regions reporting near-complete blockage of influenza virus circulation [42]. This reduction was the result of increased travel restrictions, greater attention to personal hygiene, and widespread mask-wearing, which together curtailed the spread of all respiratory viruses, including influenza [70].

A study by [74] found that influenza detection rates decreased by more than 90% during the peak pandemic months relative to prior years. This evidence underscores the effectiveness of NPIs against influenza and suggests that such measures should be employed boldly in future outbreaks to control transmission and severity.

#### 4.1.2. Public Health Measures and Reduced Transmission

The decrease in influenza cases during the COVID-19 pandemic can be accurately attributed to public health actions aimed at containing the spread of SARS-CoV-2. Social distancing, school cancellations, promoting handwashing, and wearing face masks are all distinct facets of life, yet they worked in harmony to reduce the rate of reported influenza cases [43].

The success of these measures in curtailing influenza cases has raised the debate about the possibility of implementing more focused non-pharmaceutical interventions (NPIs) in future influenza seasons. The indication by [44] that masking in high-risk settings, such as healthcare facilities and public transport, could reduce influenza circulation without severe societal restrictions is helpful. Furthermore, the results suggest that greater attention to sanitation behaviour may serve as an enduring control for the spread of influenza.

The epidemiological and cardiovascular shifts observed across the pre-COVID-19, pandemic, and post-COVID-19 periods are summarised in Table 3. This comparison highlights how public health interventions, population immunity, and viral co-circulation have reshaped both influenza dynamics and associated cardiovascular risk.

### 4.2. COVID-19 and Cardiac Complications

In concordance with the pandemic that emerged during the COVID-19 period, various cardiovascular complications have been observed due to the widespread spread of the SARS-CoV-2 virus [48]. These heart complications include acute cases such as myocarditis and ACS, as well as long-term health issues related to the heart.

#### 4.2.1. COVID-19-Induced Myocarditis and Acute Coronary Syndrome (ACS)

Myocardial harm has been associated with a SARS-CoV-2 infection in the form of myocarditis or ACS [49]. The tissues of the heart can be directly affected when a virus binds to the ACE2 receptors present in the body/which feed angiotensin. This causes increased heart inflammation and damage. Studies on troponin levels in COVID-19 patients have increased [50]. These patients have also shown signs of NT-proBNP peptide, with signs that also indicate heart damage occurring [51]. Other signs suggest positive acute coronary actions, while ST-segment elevation and different kinds of electrocardiogram abnormalities have also been recorded. All these factors prove the fact that patients suffering from COVID require close cardiac scrutiny, especially those who are already suffering from cardiovascular issues.

The causative agents with respect to the COVID-19-caused myocardium harm are a broad array. The hyper-worrying state associated with dire COVID can cause the strong plaque to rupture and the formation of considerations, while hypoxia can lead to respiratory issues. A direct viral infection of myocardial tissue or a systemic infection can also cause myocardial tissue damage [52].

#### 4.2.2. Thromboembolic Events and Stroke in COVID-19 Patients

COVID-19 has been linked to a higher risk of thromboembolic events (venous and arterial), including ischemic strokes [53]. Individuals with COVID-19 have a prothrombotic condition due to endothelial failure, inflammation, and hypercoagulability caused by the virus. Hospitalized COVID-19 patients are more likely to experience venous thromboembolism (VTE), including deep vein thrombosis (DVT) and pulmonary embolism (PE) [54]. COVID-19 infection increases the risk of myocardial infarction, ischemic stroke, deep venous thrombosis, and pulmonary embolism in community as well as hospital settings.

Less frequently than venous thrombotic events, arterial thrombotic events have also been described in COVID-19 patients. It was documented that arterial thromboembolic events, including MI and stroke, all increased immediately and markedly in the aftermath of SARS-CoV-2 infection in a study published in Circulation [55]. The paper notes that the virus-induced proinflammatory and prothrombotic conditions significantly increase the risk of serious thrombotic events [56]. The distinct pathways leading to COVID-19-associated thrombotic complications remain complex and have not been fully elucidated. COVID-19 virus-induced hypercoagulability predisposes to both venous and arterial thrombotic events. Patients admitted for COVID-19 had a VTE incidence of 4.5%, which was noted to be even higher among those admitted to the *ICU*s [57].

COVID-19 vastly increases the chances of further thromboembolic and ischemic strokes [58]. It is also essential to understand the other contributing factors and how to address them to improve patient outcomes.

#### 4.2.3. Mechanisms of COVID-19–Associated Thrombosis

COVID-19–associated thrombosis arises from a multifactorial interplay between endothelial injury, dysregulated inflammation, platelet activation, and coagulation pathway imbalance [54]. SARS-CoV-2 directly infects endothelial cells via angiotensin-converting enzyme 2 (ACE2) receptors, leading to endothelialitis, loss of antithrombotic surface properties, and exposure of procoagulant subendothelial matrices. This endothelial injury promotes tissue factor expression, platelet adhesion, and localised thrombin generation [75].

Systemic inflammation further amplifies thrombotic risk through cytokine-mediated activation of coagulation pathways. Elevated levels of interleukin-6, tumour necrosis factor-α, and other inflammatory mediators enhance fibrinogen synthesis, suppress endogenous anticoagulant mechanisms (including antithrombin and protein C pathways), and impair fibrinolysis. The resulting hypercoagulable milieu is reflected clinically by elevated D-dimer levels and widespread micro- and macrovascular thrombosis [54].

Platelet hyperreactivity also plays a central role. COVID-19 is associated with increased platelet activation, aggregation, and platelet–leukocyte interactions, which contribute to immunothrombosis and microvascular occlusion. In parallel, neutrophil extracellular trap formation (NET) promotes clot stabilisation and endothelial damage, further sustaining thrombogenesis [76].

Significantly, these thrombotic mechanisms extend beyond the acute phase of infection, with persistent endothelial dysfunction and coagulation abnormalities reported weeks to months after recovery. This prolonged prothrombotic state likely underpins the observed increase in post-acute risks of myocardial infarction, ischaemic stroke, and venous thromboembolism among COVID-19 survivors.

While influenza and SARS-CoV-2 share inflammatory and prothrombotic pathways, COVID-19 is distinguished by more pronounced endothelial involvement, sustained hypercoagulability, and a higher incidence of diffuse microvascular thrombosis, which may account for its greater burden of thromboembolic complications [2].

#### 4.2.4. Long-Term Cardiovascular Effects of COVID-19

The COVID-19 pandemic has presented acute health challenges while also exposing numerous cardiovascular issues that persist in survivors. Patients recovering from COVID-19 face significantly increased long-term cardiovascular risk, which remains elevated long after the post-infectious period ends. A study published in Nature Medicine investigated the extended cardiovascular outcomes among COVID-19 survivors [36]. Research findings confirm that COVID-19 survivors face a substantially higher risk of heart failure, ischemic heart disease, arrhythmia, and myocarditis compared to uninfected people, with these risks remaining elevated at least twelve months post-infection. According to research from the National Institutes of Health (NIH), unvaccinated individuals who contracted COVID-19 during the first wave demonstrated significantly higher risks for heart attack, stroke, and death that persisted up to three years post-infection [77]. The enduring impact of COVID-19 extends to cardiovascular health over extended periods.

The pathophysiological mechanisms behind such long-term effects exhibit great diversity. COVID-19 primarily affects the respiratory system but also leads to cardiac complications such as heart failure, pericarditis, and myocarditis throughout its progression [60]. Cardiac injury remains one of the most prevalent complications associated with the disease. COVID-19 long-term cardiac complications: The primary long-term cardiac complications from COVID-19 include ischemic heart disease, in addition to heart failure and arrhythmias, along with myocarditis [61]. Healthcare professionals need to conduct thorough cardiovascular screenings for all COVID-19 survivors. The population in question would experience a reduction in their long-term CVD burden through early diagnosis and timely treatment. The findings demonstrate the critical importance of vaccination and protective measures in reducing both the immediate and long-term health impacts of COVID-19.

### 4.3. Synergistic Effects of Influenza and COVID-19 on Cardiac Health

Influenza and COVID-19 pose a significant threat to cardiovascular health, as their joint effects can accelerate the onset of heart disease complications. Coinfection of SARS-CoV-2 and influenza potentiates an inflammatory response that sets off severe cardiac events such as myocardial infarction, myocarditis, and arrhythmias [63]. Influenza induces a pro-inflammatory milieu that causes both endothelial dysfunction and increased thromboembolic potential and may exacerbate COVID-19’s established prothrombotic sequelae. The cytokine storm triggered by both viruses adds to myocardial stress, which leads to a greater risk of heart failure. Understanding these synergistic effects enables the development of preventive measures such as timely immunization and early antiviral treatment, which help reduce adverse cardiovascular outcomes [64].

#### Risk of Co-Infection and Cardiovascular Complications

**Figure 8:**
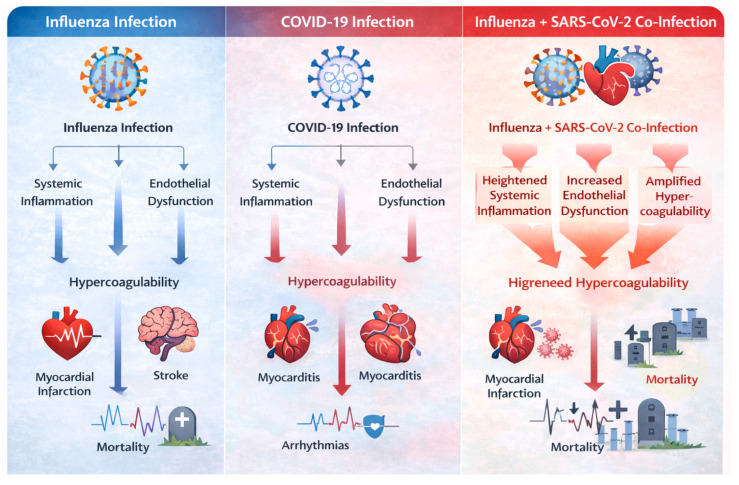
Synergistic cardiovascular injury during influenza and SARS-CoV-2 co-infection.

The risk to cardiovascular health becomes bidirectional when a person is infected with both influenza and COVID-19. In patients suffering from coinfection, increased rates of hospitalization, ICU admission, and mortality in patients with coinfection compared to single virus infections have been described [36]. Coinfection is associated with elevated systemic inflammation, resulting in higher arterial stiffness and plaque vulnerability, and hence an increased probability of ACS and stroke. In patients with co-infections, viral myocarditis becomes more severe due to additional inflammation [65]. New methods of early intervention with anticoagulation therapy and detailed hemodynamic monitoring are vital in these co-infected patients, so doctors should closely monitor cardiac symptoms. The additive and synergistic cardiovascular effects of influenza and SARS-CoV-2 co-infection are illustrated in Figure 8.

Co-infection amplifies systemic inflammation, endothelial dysfunction, and hypercoagulability beyond that observed with either virus alone, resulting in increased risk of myocardial infarction, myocarditis, arrhythmias, stroke, and cardiovascular mortality.

### 4.4. Impact of COVID-19 Vaccination on Influenza and Cardiac Health

**Figure 9:**
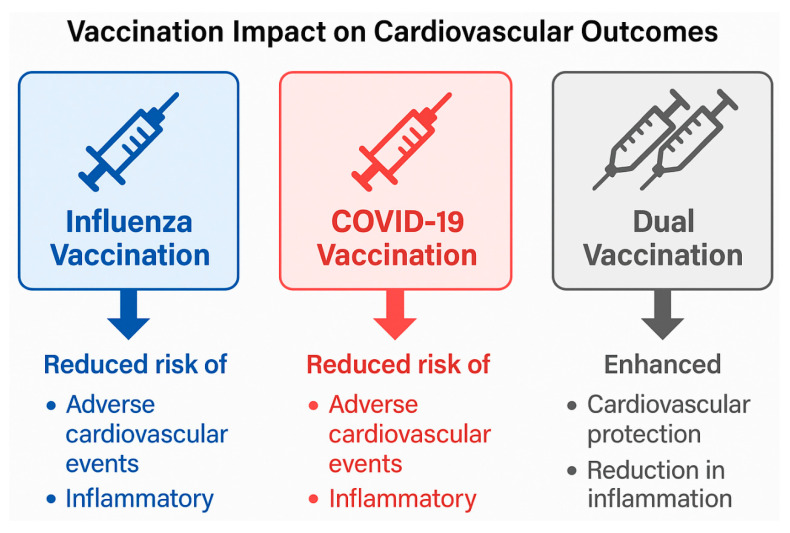
Impact of influenza, COVID-19, and dual vaccination on cardiovascular outcomes.

Because of the impact of COVID-19 vaccinations on the epidemiology of influenza and outcomes related to CVD directly and indirectly, vaccination against COVID-19 leads to a significant decrease in both severe disease from SARS-CoV-2 infection as well as the conditions of systemic inflammation, endothelial damage and thromboembolic complications caused by the viral infection of the patient to occur which leads to the increased risk for developing a cardiovascular event as a result of their disease. In patients with cardiac (CVD) disorders, the protective effects of COVID-19 vaccination have special significance since acute myocardial infarction, heart failure, arrhythmia, and stroke can often occur due to the virus [78]. The many COVID-19 vaccine deployments now being used worldwide represent a significant decrease in the cardiovascular stress experienced as a result of viruses and are changing how vaccination programs for similar respiratory illnesses, such as influenza, will be developed in the future. Figure 9 summarises the cardiovascular protective effects of influenza, COVID-19, and dual vaccination strategies, highlighting their role in reducing virus-associated inflammation, endothelial dysfunction, and adverse cardiovascular events.

Influenza and COVID-19 vaccination are each associated with a reduction in adverse cardiovascular events by attenuating systemic inflammation, endothelial dysfunction, and thrombotic risk. Dual vaccination may provide additive or synergistic cardiovascular protection, particularly in high-risk populations, by reducing cumulative inflammatory burden and preventing virus-triggered cardiovascular complications.

#### 4.4.1. Role of COVID-19 Vaccines in Preventing Influenza and Cardiac Events

COVID-19 vaccines are not influenza vaccines, but new research shows they help reduce the risk of cardiovascular complications from COVID-19. A person infected with SARS-CoV-2 experiences a significant inflammatory response, followed by changes in their blood vessels (endothelium) and clotting ability (hypercoagulable state), which increase the likelihood of developing ACS, myocarditis, and thromboembolic events. Vaccination provides some protection against severe COVID-19 and reduces viral replication; consequently, these benefits help reduce downstream cardiovascular risks associated with COVID-19 disease [68].

Recent information indicates that vaccinated patients have the potential for lower rates of CVD after recovering from infection compared to unvaccinated patients; therefore, vaccinated patients are less likely than unvaccinated patients to experience coronary artery blockade (myocardial infarction), worsening of heart failure, or stroke following infection. This is especially relevant for patients with pre-existing CVD, who are at even higher risk of complications from respiratory viral infection; thus, a reduction in hospitalisation rates associated with COVID-19 will benefit many patients with this condition by reducing the burden on healthcare systems and allowing better management of influenza-related cardiology complications during times of peak respiratory virus activity [69].

Additionally, the combination of SARS-CoV-2 and influenza vaccines may yield enhanced protective effects via a mechanism (combination of the two vaccines) to reduce coincident active virus-initiated inflammatory pathways, thereby decreasing the potential risk of co-infection. Thus, vaccination against SARS-CoV-2 and influenza together would reduce the total cardiovascular impact of sequential or concomitant infections with both viruses [69]. The information in this study supports integrated vaccination strategies for SARS-CoV-2 and influenza, especially among individuals at increased risk for cardiovascular events.

#### 4.4.2. Safety and Efficacy of Dual Vaccination for COVID-19 and Influenza

There is a wealth of data evaluating the safety and efficacy of simultaneously administering both COVID-19 and influenza vaccines; extensive observational studies and surveillance data from both pre- and post-marketing studies suggest that receiving both vaccines at the same time does not raise an individual’s risk for experiencing serious adverse events (SAEs) (for example, myocarditis, arrhythmias, thromboembolic complications) above what would be expected from each vaccine alone [79].

Most adverse events associated with receiving both vaccines at once are mild in intensity and temporary in duration, including injection-site pain, fatigue, and a mild fever. Also, co-administering COVID-19 and influenza vaccines preserves the immunogenicity of both vaccines, as evidenced by robust antibody responses against SARS-CoV-2 and various influenza strains.

Dual vaccination is of particular concern to older adults and patients with CVD, where the uptake of vaccines has generally been low due to issues with access and perception of safety. Offering dual vaccination is likely to increase vaccine uptake, training, and adherence, and ultimately, provide greater protection against virus-related cardiovascular problems.

Dual vaccination strategies represent a practical and cost-effective method for reducing seasonal spikes in hospitalisations and cardiovascular complications. The current body of evidence indicates that co-administering COVID-19 and influenza vaccines should be a cornerstone of preventive care management for patients at risk of developing CVD or establishing CV risk factors [80].

Given the growing body of evidence linking vaccination to reduced cardiovascular morbidity, it is essential to synthesise findings across influenza, COVID-19, and dual-vaccination strategies. Table 4 summarises key clinical studies evaluating the cardiovascular benefits and safety of these vaccination approaches, particularly in high-risk populations.

### 4.5. Changes in Clinical Management and Protocols

The COVID-19 pandemic precipitated profound changes in healthcare delivery, particularly in the management of CVD. The need to balance infection control with continuity of care led to rapid adaptations in clinical protocols, resource allocation, and patient monitoring strategies. Many of these changes have persisted into the post-COVID era and continue to influence the management of influenza-related cardiac complications.

#### 4.5.1. Adaptations in Cardiac Care During the COVID-19 Pandemic

During the COVID-19 pandemic, healthcare systems around the globe changed how they cared for patients with cardiovascular conditions to reduce the risk of virus transmission while still providing the critical services needed. Hospitals cancelled elective surgeries and implemented a prioritisation framework to ensure that patients with more severe cardiac conditions, such as ACS, decompensated heart failure, and life-threatening arrhythmias, were treated promptly. Triage systems that included multiple specialties helped identify and classify patients by cardiovascular risk and allocate resources more efficiently to treat them [83].

The development of clinical protocols that included a greater focus on assessing patients for potential cardiac injury caused by viruses and, as a result, evaluate routinely cardiac biomarkers and performing imaging studies in patients with respiratory infections has helped identify myocarditis, thromboembolic events, acute heart failure, and other complications developing as a consequence of a viral infection earlier in their course of development [72]. Consequently, refined clinical protocols from the pandemic period have increased preparedness for treating patients who develop cardiac complications due to influenza, especially during periods of influenza outbreaks or high influenza virus circulation.

#### 4.5.2. Use of Telemedicine and Remote Monitoring

In the COVID-19 period, one of the most significant changes to cardiovascular care has been the rapid adoption of telehealth and remote patient monitoring (RPM) technologies. Virtual consultations were a key part of continuing to provide high-quality healthcare to patients with chronic CVDs, allowing patients to reduce their risk by reducing the number of visits to healthcare facilities, avoiding unnecessary exposure to COVID-19, and decreasing the burden of travelling to receive care [73]. Remote patient monitoring technologies, such as smart watches and home blood pressure/rhythm monitors, have enabled early identification of patients who are clinically deteriorating.

For patients recovering from viral illnesses such as influenza and COVID-19, telehealth enables timely follow-up appointments, medication adjustments, and patient education, thereby reducing hospital readmissions and improving outcomes. Telehealth’s importance will not diminish as we enter the post-COVID-19 phase of healthcare delivery and will remain a key component of the future of cardiovascular care [73].

#### 4.5.3. Resource Allocation and Intensive Care Unit Reorganization

The huge demand on the healthcare system during the COVID-19 pandemic necessitated major reorganisation of ICUs and critical supplies [84]. Many Cardiovascular ICUs were converted into ICUs for patients with severe respiratory failure, necessitating flexible staffing and cross-training across all disciplines. This highlighted the interdependence of respiratory and cardiovascular care during viral outbreaks [74].

As a result, many hospitals developed scalable ICU models and surge capacity plans that incorporated input from cardiovascular specialists into their pandemic preparedness strategies. These models have improved the management of severe flu cases complicated by acute cardiac events, such as cardiogenic shock or desynchronised arrhythmia. The increased collaboration between cardiology, intensive care, and infectious disease teams has created a permanent legacy from the pandemic that will strengthen healthcare preparedness for future outbreaks of respiratory viruses [74].

## 5. Long-Term Cardiac Consequences of Influenza and COVID-19

### 5.1. Post-Viral Myocarditis and Long-Term Cardiac Health

Myocarditis, an inflammatory heart muscle disease, is a known complication of viral infections such as influenza and COVID-19. Influenza-related myocarditis is not usual, although it can lead to fatal cardiac dysfunction, such as left ventricular dysfunction and arrhythmia. Evidence suggests that patients with viral myocarditis may develop dilated cardiomyopathy, leading to long-term cardiac functional disabilities and a heightened risk of heart failure [44].

COVID-19, on the other hand, has greater potential to cause myocarditis because it infects cardiomyocytes directly through angiotensin-converting enzyme 2 (ACE2) receptors [85], leading to chronic inflammation and fibrosis. Post-COVID myocarditis was also reported in asymptomatic or mildly symptomatic patients, indicating increased long-term cardiovascular risk [36]. The persistence of myocardial inflammation in these patients is a concern for chronic heart dysfunction, necessitating long-term monitoring and cardio protective measures.

### 5.2. Chronic Cardiovascular Sequelae After Influenza Infection

Exacerbation of underlying CVD, acute myocardial infarction (AMI) [12], and stroke are all linked with influenza infections. Endothelial dysfunction, plaque destabilization, and increased thrombotic activity are consequences of influenza-induced pro-inflammatory responses that can lead to detrimental cardiovascular complications. The risk of AMI is six times greater in the first week after an influenza illness, according to epidemiological research [86].

Longitudinal research also demonstrates that people who have been hospitalized with severe influenza illness are at elevated risk of CVD death for years after the disease. Chronic inflammatory responses and subclinical cardiac injury are most likely to account for this extended impact. By reducing the severity of illness and systemic inflammation, yearly influenza vaccination has been found to lower these dangers [87].

#### Short-Term Versus Long-Term Cardiovascular Risk Following Influenza Infection

Influenza infection is associated with a temporally distinct pattern of cardiovascular risk, characterised by pronounced short-term vulnerability, followed by a more modest but persistent long-term risk.

Short-term cardiovascular risk is most prominent during the acute phase of infection and the immediate post-infectious period. Epidemiological studies consistently demonstrate a marked increase in acute myocardial infarction, ischaemic stroke, heart failure exacerbation, and arrhythmias within the first 7 days following laboratory-confirmed influenza infection. This early risk is driven predominantly by systemic inflammation, endothelial dysfunction, plaque destabilisation, hypercoagulability, and increased myocardial oxygen demand, resulting in a well-defined temporal clustering of cardiovascular events [27].

In contrast, long-term cardiovascular risk following influenza appears to be lower in magnitude but clinically significant, particularly among individuals with severe infection or pre-existing cardiovascular disease. Longitudinal cohort studies suggest that patients hospitalised with influenza may experience an elevated risk of cardiovascular mortality and heart failure over the following months or years. Proposed mechanisms include residual inflammatory activity, subclinical myocardial injury, persistent endothelial dysfunction, and acceleration of underlying atherosclerotic processes [36].

Notably, while short-term influenza-associated cardiovascular risk is acute, intense, and temporally constrained, long-term risk reflects a chronic disease-modifying effect that may contribute to sustained cardiovascular vulnerability. This temporal distinction has direct implications for clinical management, highlighting the importance of early antiviral therapy and acute cardiovascular surveillance during infection, as well as longer-term risk factor optimisation and preventive strategies, including vaccination [27,36]. Table 5 summarises the key differences between short-term and long-term cardiovascular risk following influenza infection, highlighting variations in timing, clinical events, underlying mechanisms, and preventive implications.

### 5.3. Post-COVID Cardiovascular Syndromes: Long COVID and Post-Acute Sequelae

For conceptual clarity, it is essential to distinguish between post-acute COVID-19 and Long COVID [88], terms that are sometimes used interchangeably in the literature. Post-acute COVID-19 broadly refers to the persistence or emergence of symptoms beyond the acute phase of SARS-CoV-2 infection, typically lasting more than 4 weeks after the initial illness. Within this spectrum, Long COVID, also referred to as post-acute sequelae of SARS-CoV-2 infection (PASC), is a defined clinical entity characterised by symptoms lasting ≥12 weeks that alternative diagnoses cannot explain. Long COVID frequently involves multisystem manifestations, including persistent cardiovascular abnormalities such as autonomic dysfunction, arrhythmias, exertional intolerance, and microvascular injury. In this section, the term Long COVID is used to describe sustained cardiovascular sequelae that meet established duration-based definitions, whereas post-acute COVID-19 is used in a broader temporal context.

The cardiovascular effects of COVID-19 are not confined to acute infection; a considerable percentage of survivors continue to experience symptoms referred to as “Long COVID” or post-acute sequelae of SARS-CoV-2 infection (PASC) [68]. Cardiovascular complications associated with long-term COVID-19 include autonomic dysfunction, palpitations, exertional intolerance, chronic dyspnea, and postural orthostatic tachycardia syndrome (POTS). Recent cohort studies indicate that, up to one year after infection, COVID-19 survivors are more likely to develop significant adverse cardiovascular complications, including heart failure, ischemic heart disease, thromboembolism, and arrhythmias [5]. The persistence of these effects is attributed to a chronic hyperinflammatory syndrome, endothelial damage, and microvascular dysfunction. For COVID-19 survivors, regular cardiac screening and early intervention are necessary given the prevalence of long-term cardiovascular dysfunction.

Although a growing body of literature has documented cardiovascular manifestations of long COVID [89,90,91], including myocardial injury, arrhythmias, thromboembolic events, and heart failure, the existing evidence remains heterogeneous, with substantial gaps in long-term prospective follow-up, mechanistic elucidation, and comparative analyses across viral respiratory infections. In particular, robust longitudinal studies distinguishing transient post-infectious cardiovascular dysfunction from persistent structural or inflammatory cardiac injury remain limited, and direct comparisons between post-influenza and post-COVID cardiovascular sequelae are scarce.

Persistent Thrombotic and Hypercoagulable States after COVID-19 Recovery.

Emerging evidence indicates that a subset of patients recovering from COVID-19 exhibits sustained hypercoagulability and thrombogenic risk extending beyond the acute infection phase [92]. Post-acute COVID-19 has been associated with persistent endothelial dysfunction, elevated D-dimer levels, platelet hyperreactivity, and ongoing activation of coagulation pathways, even in individuals without severe acute illness. Mechanistically, residual endothelial injury, immune dysregulation, circulating pro-inflammatory cytokines, and dysregulated fibrinolysis have been implicated in maintaining a prothrombotic milieu after viral clearance [75].

Notably, clinical and laboratory studies have reported delayed venous and arterial thrombotic events, including pulmonary embolism, ischemic stroke, and microvascular thrombosis, occurring weeks to months following apparent recovery. These findings support the concept that post-COVID cardiovascular risk is not solely driven by myocardial injury or autonomic dysfunction but also by prolonged haemostatic imbalance. While routine long-term anticoagulation is not universally recommended, these observations highlight the importance of risk stratification, close follow-up, and consideration of thromboprophylaxis in selected high-risk post-COVID patients, particularly those with pre-existing CVD or prior thromboembolic events [75].

### 5.4. Influenza vs. COVID-19: Long-Term Outcome Comparisons

Chronic cardiovascular complications are the result of both COVID-19 and influenza, even though there are considerable differences in their mechanisms and clinical effects. COVID-19 would seem to have more severe and long-lasting cardiovascular effects than influenza, which is mainly thrombogenic effects and acute inflammatory reactions that lead to cardiac complications [93].

COVID-19, in contrast to influenza, has a greater potential to cause myocarditis, endothelial dysfunction, and a hypercoagulable state, thus favouring thromboembolic events [55]. Furthermore, Long COVID patients had more prolonged symptom duration, as well as cardiovascular presentations, in contrast to post-influenza [86]. In contrast to influenza, which tends to be seasonal and drives immunization calendars, the long-term cardiovascular consequences of COVID-19 require continued research and specialized management protocols.

**Figure 10:**
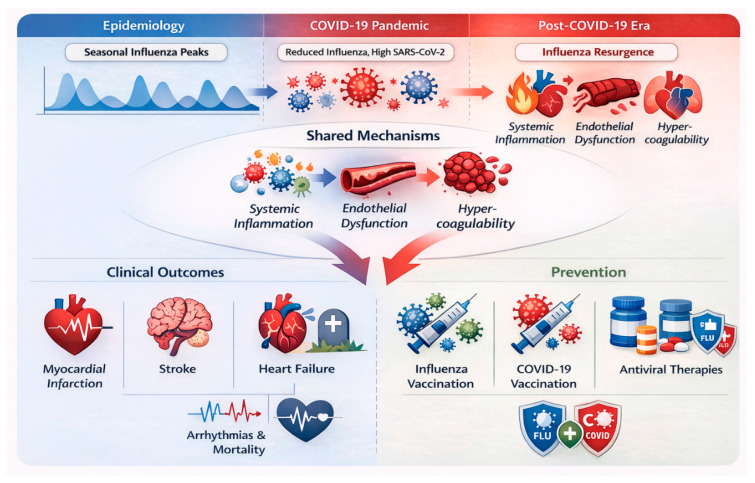
Integrated overview of influenza-related cardiovascular risk across the pre- and post-COVID-19 eras [94].

COVID-19 is showing an increased load of chronic CVD, highlighting the need for ongoing surveillance, prevention, and early intervention measures, even though both viral infections are serious risks to cardiovascular health [36]. Comparative post-viral cardiac dysfunction studies will be crucial for the sake of refining treatment protocols and reducing long-term cardiovascular morbidity. An integrated synthesis of epidemiological trends, shared pathophysiological mechanisms, clinical cardiovascular outcomes, and preventive strategies across the pre- and post-COVID-19 eras is presented in Figure 10.

Seasonal influenza, SARS-CoV-2, and CVD are all interconnected over time, as shown in the schematic. Before the onset of COVID-19, predictable patterns related to the seasonal circulation of influenza corresponded with cyclical peaks of myocardial infarction (heart attack), stroke, exacerbations of heart failure, and cardiovascular mortality. The pandemic significantly suppressed influenza transmission through extensive public health measures (e.g., stay-at-home orders), thereby modifying population immunity levels and altering patterns of cardiovascular risk during COVID-19 [94]. Following the end of the COVID-19 pandemic, there will be a resurgence of influenza, with residual inflammatory and endothelial vulnerabilities (from SARS-CoV-2 infection) that may increase cardiovascular risk. Thus, mechanisms responsive to viral infection drive systemic inflammation, endothelial dysfunction, increased coagulability, and imbalances in oxygen demand and supply, leading to adverse cardiovascular reactions. Additionally, preventive strategies are available to reduce the incidence of virus-triggered cardiovascular morbidity and mortality, such as influenza vaccines, COVID-19 vaccines, antiviral therapies (for both viruses), and CVD risk management strategies assessed throughout both the pandemic and post-pandemic era [69].

Hospitalisation for acute respiratory viral infections is increasingly recognised as a trigger for both new-onset heart failure and exacerbation of pre-existing heart failure. Extensive population-based studies have demonstrated elevated risks of incident heart failure following COVID-19 hospitalisation, with risk persisting beyond the acute phase. In contrast, influenza-related hospitalisation has long been associated with acute decompensation due to increased metabolic demand, myocardial inflammation, and fluid shifts. Comparative analyses suggest that although both infections increase heart failure risk, COVID-19 is more frequently associated with sustained myocardial injury and long-term ventricular dysfunction. In contrast, influenza-related heart failure events tend to cluster temporally around the acute illness. These distinctions have important implications for post-discharge cardiovascular surveillance and secondary prevention strategies.

## 6. Discussion

### 6.1. Summary of Key Findings

**Figure 11:**
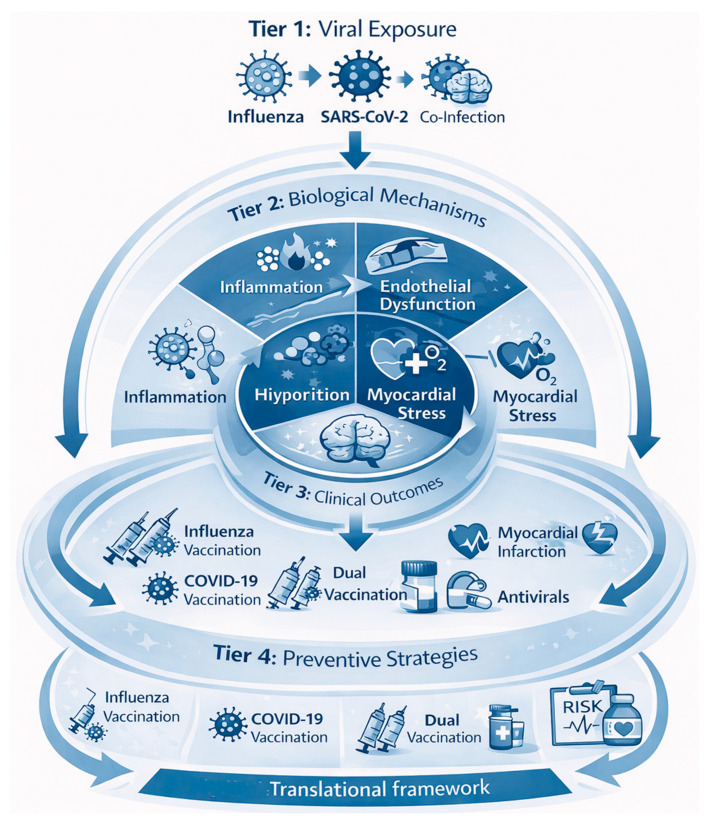
Translational framework linking viral respiratory infections to cardiovascular outcomes and prevention strategies.

The current research investigated the cardiovascular complications of seasonal influenza both before and after the COVID-19 pandemic. The study emphasizes the widespread effect of influenza-related cardiovascular morbidity and death, with an emphasis on the interplay of viral infections and acute cardiac problems such as stroke, myocardial infarction, and myocarditis. Furthermore, the influence of the COVID-19 pandemic on influenza epidemiology and its potential long-term cardiovascular effects was evaluated, resulting in substantial alterations in illness patterns and health outcomes. The epidemiological associations and mechanistic pathways discussed in this review are synthesised in Table 1, Table 2 and Table 3, while the preventive impact of vaccination strategies on cardiovascular outcomes is summarised in Table 4. The interconnection between viral infection, cardiovascular injury, and preventive strategies can be conceptualised as a translational continuum linking biological mechanisms to clinical and public health interventions (Figure 11).

To ensure interpretive clarity, it is essential to differentiate conclusions drawn from pre-COVID-19 evidence from those informed by post-COVID-19 observations. Associations between seasonal influenza and acute cardiovascular events, including myocardial infarction, stroke, heart failure exacerbation, and cardiovascular mortality, are primarily supported by robust pre-COVID-19 epidemiological studies, self-controlled case series, and cohort analyses conducted under stable influenza circulation patterns.

Conversely, conclusions about altered influenza epidemiology, modified cardiovascular risk profiles, impacts on healthcare systems, viral co-infections, and long-term cardiovascular vulnerability are predominantly based on post-COVID-19 observational data, surveillance reports, and emerging longitudinal studies. Findings from the post-COVID-19 period should therefore be interpreted within the context of residual confounding factors such as public health interventions, changes in population immunity, and healthcare service disruptions, and presented as evolving trends rather than definitive causal inferences.

Respiratory viral infections, such as influenza and SARS-CoV-2, activate inflammatory and thrombotic processes that lead to both short-term and long-term cardiovascular problems. Preventive measures, especially vaccination, early antiviral treatment, and optimising cardiovascular risk factors, disrupt this cascade and create essential opportunities to mitigate virus-related cardiovascular morbidity and mortality in the post-COVID-19 period.

Influenza and Cardiovascular Complications: There is a wealth of clinical and epidemiological evidence supporting the link between seasonal flu and cardiovascular problems. Flu-induced inflammation and coagulation problems increase the risk of heart failure, stroke, and acute myocardial infarction [95]. Patients with pre-existing cardiovascular illness are more likely to experience morbidity as a result of influenza infection, according to studies. The pathophysiologic routes include viral invasion of the heart, a systemic inflammatory response, and enhanced platelet aggregation, all of which result in thrombotic events [35].

Impact of the COVID-19 Pandemic on Influenza Trends: The COVID-19 pandemic led to an abrupt reduction in influenza cases globally due to public health interventions such as mask-wearing, social distancing, and movement restrictions. Rebound influenza in the post-pandemic era has been accompanied by altered transmission patterns and increased severity in some locations, possibly due to weakened population immunity [82]. Such epidemiologic changes in influenza necessitate a reassessment of vaccine strategy and public health preparedness.

Influenza and COVID-19 Synergistic Effect on Cardiovascular Health: The study demonstrates that COVID-19 and influenza may operate synergistically to aggravate CVD. Two-virus co-infection has been linked to higher hospitalization rates, admissions to *ICU*s, and death. Both viruses cause a hyper inflammatory reaction, increasing the patient’s risk of thromboembolic consequences, endothelial damage, and cardiac infarction [71]. Understanding these relationships is essential for developing tailored preventive treatments, such as early vaccines and therapies.

Long-Term Cardiovascular Consequences: COVID-19 and influenza have both been linked to long-term cardiovascular complications. Post-viral myocarditis, autonomic dysfunction, and the risk of thromboembolic events have all been observed in patients recovering from both infections. Post-COVID syndrome (Long COVID) has been linked to chronic cardiovascular problems such as arrhythmias, heart failure, and arterial stiffness [3,82]. These findings highlight the importance of long-term cardiovascular follow-up in post-viral patients.

Preventive Measures with Clinical Implications: The study supports the use of influenza and COVID-19 vaccinations to prevent cardiovascular problems. There is evidence that influenza vaccination not only lessens the impact of respiratory infections but also lowers the risk of abrupt cardiac events [82]. Vaccination, however, remains underused, especially in high-risk populations. Increasing vaccination rates and including cardiovascular risk factor evaluation in influenza management plans could considerably enhance patient outcomes [37].

### 6.2. Integration with Existing Literature and Comparative Interpretation

While earlier sections of this review describe epidemiological trends, mechanisms, and clinical outcomes of influenza-associated cardiovascular complications, this Discussion integrates these findings within the broader context of existing literature and recent post-pandemic evidence.

Numerous extensive population-based studies and self-controlled case series have consistently demonstrated a marked increase in acute myocardial infarction and stroke risk within days to weeks following laboratory-confirmed influenza infection [10,13,96]. The magnitude of risk observed in this review, particularly the approximately six-fold increase in myocardial infarction risk within seven days [12], is concordant with findings reported in prior landmark studies from Canada, the United Kingdom, and Scandinavia. These convergent observations across diverse populations strengthen the causal inference between influenza infection and acute cardiovascular events.

Compared with pre-COVID-19 literature, post-pandemic studies suggest that influenza-associated cardiovascular risk may be amplified or temporally shifted due to altered population immunity and co-circulation with SARS-CoV-2 [2]. While predictable winter peaks in cardiovascular events characterised earlier influenza seasons, recent reports indicate more heterogeneous seasonal patterns, complicating surveillance and prevention strategies. This evolution highlights an essential departure from historical influenza–cardiovascular paradigms and underscores the need for adaptive public health planning [38,85].

Mechanistically, the inflammatory and prothrombotic pathways identified in this review align closely with established models of infection-triggered plaque destabilisation [56]. However, emerging post-COVID-19 data suggest that residual endothelial dysfunction and immune dysregulation following SARS-CoV-2 infection may potentiate influenza-related cardiovascular injury beyond levels previously observed [28,92]. This layered inflammatory burden represents a novel conceptual contribution of the current review.

In contrast to earlier reviews that primarily focused on either influenza or COVID-19 in isolation [2,3,71,95,97,98], this paper provides an integrated synthesis of pre- and post-pandemic evidence, highlighting how changes in viral epidemiology, vaccination behaviour, and healthcare delivery intersect to shape contemporary cardiovascular risk. By situating influenza-associated cardiovascular complications within this evolving landscape, the present Discussion extends prior work. It offers clinically relevant insights for risk stratification, vaccination policy, and long-term cardiovascular surveillance.

### 6.3. Strengths and Limitations of the Review

This review has several important strengths. First, it provides a comprehensive, comparative synthesis of influenza-associated cardiovascular complications across the pre-COVID-19, pandemic, and post-pandemic eras, integrating epidemiological evidence with mechanistic and clinical insights. Unlike prior influenza-focused reviews, this study explicitly contextualizes cardiovascular risk within the evolving landscape shaped by SARS-CoV-2, including altered influenza epidemiology, residual endothelial dysfunction, and long-term post-viral cardiovascular vulnerability [2]. Second, the review adopts a translational framework that links biological mechanisms to clinical outcomes and preventive strategies, highlighting vaccination and early antiviral therapy as modifiable interventions that can reduce virus-triggered cardiovascular events. Third, the manuscript integrates evidence from diverse study designs, including self-controlled case series, population-based cohorts, and randomized trials, thereby strengthening the consistency and biological plausibility of the observed associations.

Several limitations should also be acknowledged. As a narrative review, this study does not generate new primary data, and causal inferences cannot be definitively drawn. Much of the available evidence linking influenza and SARS-CoV-2 infection to cardiovascular outcomes comes from observational and registry-based studies, which are susceptible to residual confounding, misclassification of viral exposure, and temporal bias. In addition, heterogeneity in study populations, outcome definitions, and follow-up duration limits direct quantitative comparison across studies. Although emerging data suggest increased risks of new-onset heart failure, heart failure exacerbations, and post-acute cardiovascular sequelae following hospitalization for influenza and COVID-19, particularly among older adults and individuals with pre-existing cardiovascular disease, robust long-term prospective studies directly comparing these viral infections remain limited.

Furthermore, although the respiratory syncytial virus and other seasonal respiratory pathogens may also contribute to cardiovascular morbidity, particularly in vulnerable populations, they were not examined in depth to maintain a focused scope on influenza and SARS-CoV-2. Similarly, metabolic and oncologic outcomes associated with COVID-19 were beyond the cardiovascular emphasis of this review and were therefore not systematically addressed. Future longitudinal, multi-pathogen studies integrating cardiovascular, metabolic, and inflammatory outcomes will be essential to more fully characterize the long-term cardiovascular consequences of respiratory viral infections.

### 6.4. Research Gaps and Areas for Future Study

Despite a substantial body of work elucidating the cardiovascular impacts of seasonal influenza and COVID-19, significant knowledge gaps remain. Bridging these gaps is essential for developing improved treatment guidelines, vaccination strategies, and health outcomes for people living with viral infections. The following areas should be investigated:

#### 6.4.1. Long-Term Cardiovascular Outcomes

A lack of systematic, longitudinal studies assessing the cardiovascular effects of influenza and COVID-19 exists. The majority of the research focuses on the acute consequences, and as a result, there is a gap in understanding the persistence and progression of cardiovascular dysfunction after an infection. Future studies should address the long-term effects of viral infection on cardiovascular outcomes, including arrhythmias, heart failure, and thromboembolic events.

#### 6.4.2. Mechanisms of Viral-Induced Cardiovascular Damage

Inflammation, endothelial dysfunction, and hypercoagulability have all been suggested to play a role in both the flu and COVID-19, but the molecular pathways and immune responses remain far from elucidated. There is a need for further research to characterize the individual contributions of each virus to myocardial injury, vascular inflammation, and chronic cardiovascular events. Understanding these mechanisms will help in developing targeted therapies.

#### 6.4.3. Co-Infection and Cardiac Health

Cardiovascular sequelae of flu and COVID-19 coinfection remain unknown. Given the potential for co-infection, studies must examine whether co-infection increases cardiac morbidity or changes the disease course. Exploring the synergistic effect of combined infection will provide us with significant knowledge to control and prevent the development of severe cardiac complications in involved patients.

#### 6.4.4. Efficacy of Vaccination in Reducing Cardiac Events

Although flu and COVID-19 vaccines have been shown to reduce the severity of respiratory disease, their impact on cardiovascular risk has yet to be assessed. Research is also needed to determine if vaccination actually lessens post-viral cardiac sequelae, specifically among high-risk populations who have underlying CVD [99]. The feasibility of using combined vaccination schemes will also be helpful for future public health policy.

Post-Viral Syndrome and Cardiac Rehabilitation. Both Long COVID and post-flu syndromes have cardiovascular effects that last, but there are no standard guidelines for rehabilitation strategies for viral myocarditis, autonomic dysfunction, and persistent cardiovascular impairment. There is a critical need to improve patient outcomes by developing evidence-based rehabilitation protocols for post-viral cardiac rehabilitation.

#### 6.4.5. Healthcare Policy and Surveillance Strategies

Standardized long-term cardiovascular monitoring programmes for patients who have recovered from severe influenza or COVID-19 are unavailable. Future studies should focus on surveillance-based systems and policy actions to lower long-term cardiovascular risk in exposed populations. Emphasis on robust construction of the healthcare infrastructure for long-term surveillance and early interventions will play a crucial role in diminishing the disease burden.

## 7. Conclusions

The connection between influenza and cardiovascular complications has been established for many years. Yet, growing evidence now shows heightened risk of acute myocardial infarction, myocarditis, stroke, and heart failure following influenza epidemics [2]. COVID-19’s pandemic shifted paradigms while introducing novel obstacles to viral epidemiology and CVD management, as well as to clinical interventions. The simultaneous presence of SARS-CoV-2 [38] and influenza viruses amplifies systemic inflammation, endothelial dysfunction, and prothrombotic conditions, leading to a higher occurrence of severe cardiovascular complications [48].

The post-COVID era demands new public health strategies that encompass vaccination policy and the distribution of health resources. The temporary suppression of influenza activity by pandemic-control, non-pharmaceutical interventions necessitates ongoing caution and mitigation given its renewed circulation. The necessity to overhaul cardiovascular risk management stems from COVID-19’s long-term effects, which increase myocarditis [61], arrhythmia, and thromboembolism risks [58].

Future research must examine the mechanisms linking viral infection to heart disease, while also improving preventive measures such as double-vaccination campaigns. Treatment recommendations for flu and COVID-19 must address cardiovascular complications to reduce mortality and the long-term effects of the illness. The development of a comprehensive, evidence-based strategy is essential to minimize the long-term cardiovascular effects of respiratory virus infections. Direct head-to-head comparisons of post-acute cardiovascular sequelae following hospitalisation for COVID-19 versus influenza remain limited and represent an important area for future research.

## Figures and Tables

**Table 1:** Cardiovascular complications associated with influenza infection.

Cardiovascular Outcome	Evidence Type	Magnitude of Risk	Proposed Mechanism	References
AMI	Self-controlled case series, observational cohorts	~6-fold increased risk within 7 days of laboratory-confirmed influenza	Systemic inflammation, plaque destabilisation, endothelial dysfunction, hypercoagulability	[11,12,13]
Ischaemic Stroke	Systematic reviews, case–control studies	OR 2.4–2.9 within 1 week–1 month post-infection	Pro-thrombotic state, endothelial injury, cytokine-mediated vascular instability	[11,20,21]
Heart Failure Exacerbation	Population-based cohorts	Increased hospitalisation and mortality during influenza seasons	Increased metabolic demand, myocardial inflammation, and fluid overload	[14,15,16]
Arrhythmias	Clinical observational studies	Increased incidence during acute infection	Sympathetic activation, myocardial inflammation, hypoxia	[17]
Cardiovascular Mortality	Time-series and ecological studies	Seasonal peaks aligned with influenza epidemics	Systemic inflammation, thrombogenesis, plaque rupture	[18]
Reduction with Influenza Vaccination	Meta-analyses, RCTs	~34% reduction in major adverse cardiovascular events	Reduced viral replication, attenuated inflammatory response	[19,22]

*Note: AMI = acute myocardial infarction; OR = odds ratio; RCT = randomised controlled trial.*

**Table 2:** Mechanisms of cardiovascular injury induced by influenza and SARS-CoV-2.

Pathophysiological Mechanism	Influenza Virus	SARS-CoV-2	Cardiovascular Consequences
Direct myocardial invasion	Rare but documented myocarditis	Frequent via ACE2 receptor binding	Myocarditis, reduced ventricular function
Endothelial dysfunction	Cytokine-mediated endothelial activation	Direct endothelial infection and inflammation	Plaque instability, thrombosis
Systemic inflammation	IL-6, TNF-α-driven cytokine response	Severe cytokine storm in critical illness	ACS, arrhythmias, heart failure
Hypercoagulability	Platelet activation, increased fibrinogen	Marked prothrombotic state	MI, stroke, venous thromboembolism
Oxygen supply–demand mismatch	Fever, tachycardia, hypoxia	Severe hypoxia and respiratory failure	Type 2 MI, arrhythmias
Long-term cardiac sequelae	Increased long-term CVD mortality	Persistent risk ≥12 months post-infection	Heart failure, arrhythmias, ischemic heart disease

*Note: This table presents an original comparative synthesis developed by the authors, integrating mechanistic evidence from multiple published studies. References [3,58] are cited as key supporting sources and do not constitute the source of the table’s structure or content.*

**Table 3:** Comparison of influenza epidemiology and cardiovascular impact before, during, and after the COVID-19 pandemic [45,46,47].

Parameter	Pre-COVID-19 Era	COVID-19 Pandemic Period (2020–2021)	Post-COVID-19 Era
Influenza incidence	Predictable seasonal peaks during the winter months	Dramatic global suppression (>90% reduction in many regions)	Rebound with altered seasonality and regional variability
Dominant drivers	Seasonal viral circulation, population immunity	Non-pharmaceutical interventions (NPIs), travel restrictions, masking	Waning population immunity, relaxed NPIs, viral co-circulation
Cardiovascular hospitalisations	Seasonal rise in MI, heart failure, and stroke	Marked reduction paralleling influenza suppression	Resurgence of influenza-related cardiac admissions
Myocardial infarction risk	~6-fold increase within 7 days of infection	Substantially reduced due to low influenza circulation	Renewed elevated risk with influenza resurgence
Stroke incidence	Increased during influenza seasons	Reduced during the pandemic	Gradual return to pre-pandemic trends
Healthcare burden	Predictable winter surges	Shifted primarily to COVID-19 care	Dual burden of influenza and COVID-19
Public health implications	Annual vaccination and surveillance	Demonstrated effectiveness of NPIs	Need for adaptive vaccination and surveillance strategies

Note: *NPIs = non-pharmaceutical interventions; MI = myocardial infarction.*

**Table 4:** Impact of influenza and COVID-19 vaccination on cardiovascular outcomes [81,82].

Vaccine Type	Study Design	Cardiovascular Outcome	Risk Reduction	Clinical Implication
Seasonal influenza vaccine	Meta-analysis of RCTs and observational studies	Major adverse cardiovascular events (MACE)	~34% reduction	Strong indication for routine vaccination in CVD patients
Influenza vaccine (post-ACS)	Randomised controlled trials	Recurrent MI and cardiovascular mortality	Greater benefit in recent ACS patients	Secondary prevention strategy
COVID-19 vaccine	Large cohort studies	MI, stroke, heart failure post-infection	Significant reduction vs. unvaccinated	Reduces long-term cardiovascular burden
Dual influenza + COVID-19 vaccination	Observational and surveillance studies	Hospitalisation and thromboembolic events	No increased adverse cardiac risk	Safe, improves uptake and protection
High-risk populations (elderly, CVD)	Population studies	All-cause and cardiovascular mortality	Consistent mortality reduction	Priority group for vaccination programmes

Note: *MACE = major adverse cardiovascular events; ACS = acute coronary syndrome.*

**Table 5:** Comparison of short-term and long-term cardiovascular risk following influenza infection.

Dimension	Short-Term Risk	Long-Term Risk
Timeframe	0–7 days (up to 30 days)	Months to years
Key events	MI, stroke, HF exacerbation, arrhythmias	HF progression, CV mortality
Mechanisms	Inflammation, thrombosis, plaque rupture	Residual inflammation, endothelial dysfunction
Risk magnitude	High, transient	Moderate, persistent
Clinical implication	Acute surveillance, antivirals	Long-term prevention, vaccination

## Data Availability

No new data were created or analyzed in this study.
